# DenHunt - A Comprehensive Database of the Intricate Network of Dengue-Human Interactions

**DOI:** 10.1371/journal.pntd.0004965

**Published:** 2016-09-12

**Authors:** Prashanthi Karyala, Rahul Metri, Christopher Bathula, Syam K. Yelamanchi, Lipika Sahoo, Selvam Arjunan, Narayan P. Sastri, Nagasuma Chandra

**Affiliations:** 1 Department of Biochemistry, Center of Research and Post Graduate Studies, Indian Academy Degree College, Bengaluru, Karnataka, India; 2 IISc Mathematics Initiative, Indian Institute of Science, Bengaluru, Karnataka, India; 3 LifeIntelect Consultancy Pvt Ltd, Marathahalli, Bengaluru, Karnataka, India; 4 Department of Biotechnology, Center of Research and Post Graduate Studies, Indian Academy Degree College, Bengaluru, Karnataka, India; 5 Department of Molecular Virology & Microbiology, Baylor College of Medicine, Houston, Texas, United States of America; 6 Department of Biochemistry, Indian Institute of Science, Bengaluru, Karnataka, India; Universita degli Studi di Pavia, ITALY

## Abstract

Dengue virus (DENV) is a human pathogen and its etiology has been widely established. There are many interactions between DENV and human proteins that have been reported in literature. However, no publicly accessible resource for efficiently retrieving the information is yet available. In this study, we mined all publicly available dengue–human interactions that have been reported in the literature into a database called DenHunt. We retrieved 682 direct interactions of human proteins with dengue viral components, 382 indirect interactions and 4120 differentially expressed human genes in dengue infected cell lines and patients. We have illustrated the importance of DenHunt by mapping the dengue–human interactions on to the host interactome and observed that the virus targets multiple host functional complexes of important cellular processes such as metabolism, immune system and signaling pathways suggesting a potential role of these interactions in viral pathogenesis. We also observed that 7 percent of the dengue virus interacting human proteins are also associated with other infectious and non-infectious diseases. Finally, the understanding that comes from such analyses could be used to design better strategies to counteract the diseases caused by dengue virus. The whole dataset has been catalogued in a searchable database, called DenHunt (http://proline.biochem.iisc.ernet.in/DenHunt/).

## Author Summary

The ‘**Den**gue **Hu**man I**nt**eraction Database’, called DenHunt, available at http://proline.biochem.iisc.ernet.in/DenHunt/ was created to catalog all interactions between dengue viral and human components published in peer-reviewed literature. There are three types of dengue-human molecular interactions in the database: direct physical interactions between dengue virus and human components, indirect interactions of human proteins affecting viral replication with no current evidence of them directly interacting with the viral components, and differentially expressed genes in dengue infected cell lines or patients. DenHunt could be used to draw network maps of human-dengue relationships which would aid in understanding dengue viral pathogenesis and such knowledge in turn can reveal new strategies for inhibiting viral replication. We also demonstrate that DenHunt could be used to compare common and diverse mechanisms of pathogenesis caused by infectious and non-infectious diseases thereby help in understanding disease mechanisms in general.

## Introduction

Dengue, an emerging infectious disease, is presently the most common arboviral disease globally. Approximately 2.5 billion people live in dengue infested regions worldwide and 390 million dengue infections are reported per year [1]. Dengue infection leads to complications ranging from mild dengue fever to more severe dengue hemorrhagic fever (DHF) and dengue shock syndrome (DSS). It is not fully understood why most patients clear dengue infections quickly without any complications, whereas others develop a potentially fatal vascular leakage syndrome or severe hemorrhages. The large size of the population prone to infection by dengue vouches for the importance of the development of vaccines for prevention and antiviral therapies to manage/treat dengue viral infections. Despite intense research efforts, no approved vaccine or antiviral therapy is yet available. Early clinical diagnosis and careful clinical management by experienced physicians and nurses to increase survival of patients are still the most commonly used strategies to treat dengue infections.

The major hindrance in understanding the host response to dengue infection and development of vaccines and antiviral therapies is the lack of an adequate animal model that can display the full spectrum of dengue immunity and disease response. Regardless of the difficulties in *in vivo* dengue viral research, significant efforts have been directed towards understanding the pathogenesis of dengue infection using *in vitro* platforms, such as cell lines as well as clinical samples such as patient’s blood, peripheral blood mononuclear cells (PBMCs) and serum. The knowledge obtained using conventional studies as well as from high-throughput technologies, such as functional genomics, transcriptomics, proteomics and yeast-two-hybrid techniques have given us important insights into the role of dengue virus interactions with its host in viral replication and pathogenesis. This valuable information remains disseminated along with other published literature in databases essentially in PubMed, making it difficult and time-consuming for dengue viral researchers to access and utilize the information for detailed computational analysis. Hence, there exists an immediate need for generating a database that provides readily usable simplified data pertaining to dengue-human interactions by collating all the existing information in literature.

Many databases have been generated for different pathogens that provide curated interactions between pathogen—host components. Specific virus databases, such as HCVpro [2] and HIV-1 Human Protein Interactions Database (HHPID) [3, 4], have been developed to host all known HCV or HIV1 –human PPIs respectively. Other databases catalogue: a) known host-viral interactions for many viruses, eg. VirHostNet 2.0 [5] and VirusMint [6] replaced by VirusMentha [7], b). host-pathogen interactions for many pathogens of viral, bacterial, fungal origin in PIG (the pathogen interaction gateway) incorporated into PATRIC [8, 9] and PHI-base [10] and c) host-pathogen interactions for many pathogens along with other intra organismal PPIs such as MINT [11], IntAct [12] and BioGRID [13]. However, dengue-human interactions are poorly represented in these databases with interactions extracted only from a few publications.

To fill this lacuna, we have developed a **Den**gue **Hu**man protein I**nt**eraction database that we call “DenHunt” which serves as a freely accessible, periodically updated comprehensive resource for the dengue research community. The objective of this study is to describe the development of the database, summarize its contents, demonstrate the complexity of the dengue-human protein interaction network and compare it with networks of proteins interacting with other pathogens or involved in other diseases. Our database is unique, compared to others as we have curated data from many publications and incorporated all genes associated with dengue viral infection into different categories such as direct interactions, indirect or functional interactions and differentially expressed genes. We show that the information from such databases can help in creating network maps of how the virus disrupts cellular signaling and immune system pathways. We also list known FDA approved drugs against the dengue virus interacting human proteins that are being used to treat various other diseases.

For each interaction in the entire interaction dataset, the National Library of Medicine (NLM) PubMed identification numbers (PMIDs) of the publication describing the interaction, Entrez Gene IDs and gene symbols of the proteins involved in the interaction, the type of patient sample or cell line used in the study, the experiments used to detect the interactions, and the viral serotype used in the study is available in the database. The database will be periodically updated and with increasing information of dengue-host interactions published in scientific literature, we expect DenHunt to attain higher coverage and curation depth and become a valuable resource for comprehensive analysis of dengue viral mechanisms and interactions, thereby enhancing our fundamental understanding of the disease.

## Methods

### Literature curation

The abstracts and publications containing original research describing association of human host proteins with dengue virus were queried using the term “dengue” or the specific dengue protein along with the keywords listed in S1 Table. These keywords were extracted from a publication that describes the construction of the HIV-1 human protein interaction database [3]. The interactions retrieved from these publications were segregated manually into direct interactions, indirect interactions and differentially expressed interactions. For a majority of abstracts, the full-text of the article was reviewed. However, in case of abstracts for which full length articles were not available, the interactions were catalogued based on the abstract alone, only if they contained complete descriptions of the interactions. Since the articles were obtained from peer-reviewed publications, all identified interactions were incorporated into the database without placing further judgment on the scientific validity of the report. Most papers describe the protein in the study as a gene synonym or alias. In this regard, the official human protein symbol and Entrez GeneID were extracted from online tools such as bioDBnet [14], Synergizer [15], GeneCards [16] and NCBI Entrez Gene database [17].

Information retrieved from relevant publications was collected and the interactions were manually catalogued into a table containing some or all of the following fields:

1. Dengue viral component: The dengue viral component involved in the interaction. This field is available only for the direct interactions.
2. Human Gene symbol: The official gene symbol of the human protein interactor.
3. Human gene Entrez ID: The gene identification numbers from Entrez Gene, NCBI's database for gene-specific information.
4. Pubmed ID: PMIDs of articles describing the interaction.
5. Patient type or Serotype: The serotype of the DENV strain or strains (DENV1, 2, 3, 4) used in the study. If clinical samples from patients such as serum, whole blood or peripheral blood mononuclear cells (PBMCs) are used, then type of patient (DF, DHF and DSS) used in the study is given.
6. System: This field gives information of the cell line used in the study if it is an *in vitro* study. If the study uses clinical samples, then the type of sample used such as serum, whole blood or peripheral blood mononuclear cells (PBMCs) is given.
7. Comparison: This field is available only for differentially expressed genes where the expression of genes in infected samples is compared with controls. eg. Dengue infected 293T cells vs uninfected 293T cells)
8. Variation: This field is available only for differentially expressed interactions. It states whether the gene is up regulated or down regulated in dengue infected samples.
9. Title: Title of the paper where the interaction was extracted.

### Database and web interface

The DenHunt database was created by integrating curated dengue and human molecular interactions and the pathways involved. The LAMP (Linux-Apache-MySQL-PHP) platform was used to develop DenHunt. The web interface was developed using BootStrap (http://twitter.github.com/bootstrap) which provides cascading style sheets framework and JavaScript functionality. The database can be primarily queried based on the dengue viral or human protein involved in the interaction. The direct interactions related to query term can be visualized as a network constructed using Cytoscape.js (js.cytoscape.org), a JavaScript based library for analysis and visualization of the network. The dataset of interactions is efficiently stored in a relational database schema. The results are stored as tables which are sortable and searchable, and allow easy access to the data of interest. The entire dataset can be downloaded as a flat file from the download section.

### Pathway enrichment analysis

We compiled a comprehensive list of human proteins which: i) interact directly with viral proteins, ii) indirectly affect dengue infection and iii) are reported to be DEGs (consistently up or down regulated in dengue infection) in at least 4 different publications in dengue infected cell lines or patients (S1 Dataset). This consolidated list was subjected to pathway analysis using the online tool WebGestalt [18, 19] and KEGG Mapper—Search Pathway tool [20]. The KEGG (Kyoto Encyclopedia of Genes and genomes) pathways enrichment was carried out by using the hypergeometric test and the P-value was adjusted by the Benjamini & Hochberg (BH) method. Only pathways that had a minimum number of 3 genes per pathway and adjP-value ≤ 0.01 were selected. We selected all the pathways identified by the KEGG Mapper—Search Pathway tool that had 20 and more dengue interacting human proteins. Enriched pathways belonging to normal biological processes were grouped into broad categories as described in the KEGG database. The broad categories and the number of genes that belong to each category are plotted as a pie chart. Pathways containing 20 or more dengue viral interacting human proteins which belong to the top 4 broad categories are plotted as a bar graph. The gene list was also queried in the KEGG Search&Color pathway to obtain graphical representations of the dengue interacting proteins in different cellular processes that the virus may target to aid its replication.

### Disease association analysis

The KEGG human disease pathways that were enriched by WebGestalt and KEGG Mapper were split into two groups: infectious and non-infectious disease group. The diseases caused by pathogens of bacterial, parasitic and viral origin were assigned to the infectious diseases group. All remaining diseases were assigned to the non-infectious disease group. An edge is placed between the gene and its associated disease and visualized as a network in Cytoscape.

### FDA approved drugs against dengue viral interacting human proteins

A subset of proteins was extracted from the entire list of dengue virus interacting human proteins where knockout, gene silencing or inhibition studies were carried out. The list of known drugs against this subset of dengue viral interacting human proteins was extracted from bioDBnet [14]. Each drug obtained was checked whether it exhibited pharmacological action against the dengue virus interacting human protein from the drug and drug target database DrugBank [21].

## Results

### Construction of the DenHunt database

Consolidation of the current knowledge on dengue published till date could help in the construction of the network of molecular events occurring during the viral life cycle. There are around 14,559 publications describing dengue viral research in PubMed till 31 October 2015. Literature describing dengue-human interactions was extracted from PubMed by queries using the keywords listed in S1 Table. The retrieved interactions were classified into three types: (i) *Direct interactions*, where the human proteins physically interact with the viral proteins or RNA, (ii) *Indirect or functional interactions*, where the human proteins affect viral replication but there exists no current evidence of them directly interacting with the viral components and (iii) *Differentially expressed interactions*, genes or proteins whose expression patterns are altered during dengue viral infection.

Out of 6576 publications retrieved after keyword search, we identified 682 direct, 382 indirect and 4120 differentially expressed interactions from 103, 151 and 41 references in PubMed respectively. Table 1 summarizes the Dengue-Human interactions catalogued from these papers. 21% of the total interactions were described in more than one paper. Of the total 4613 dengue interacting human proteins, be it direct, indirect or differentially expressed genes, 41 proteins were detected in all the three categories, and 339 were detected in two of the three categories. Data for all these three different types of interactions were compiled into three different sections in the DenHunt database, and the detailed list of all the interactions can be downloaded from the database website and is also available as S2 Dataset.

**Table 1:** Consolidated list of different types of dengue—human interactions available at the DenHunt database. The type of interactions, the total number of unique interactions for each type of interaction, the number of references that describe each type of interaction and the total number of human proteins involved in all the interactions are given in the table.

S.No	Type of Interaction	Dengue viral components	Total number of unique interactions	Number of PMID references
1	Direct Interactions of human protein with dengue viral components		682	103
		5'- and 3'-UTR, DENV RNA	31	15
		C	66	19
		E	34	14
		prM/M	24	8
		NS1	162	21
		NS2A	43	3
		NS2B	23	4
		NS3	126	19
		NS4A	15	7
		NS4B	27	5
		NS5	131	18
2	Indirect interactions through siRNA and small scale studies		382	151
3	Differentially Expressed Genes (DEGs) identified in DENV infected cell lines and patients		4120	41
	Total number of human genes or proteins involved the above interactions		4613	
	Total number of PMIDs			287

#### Direct interactions

**Fig 1:**
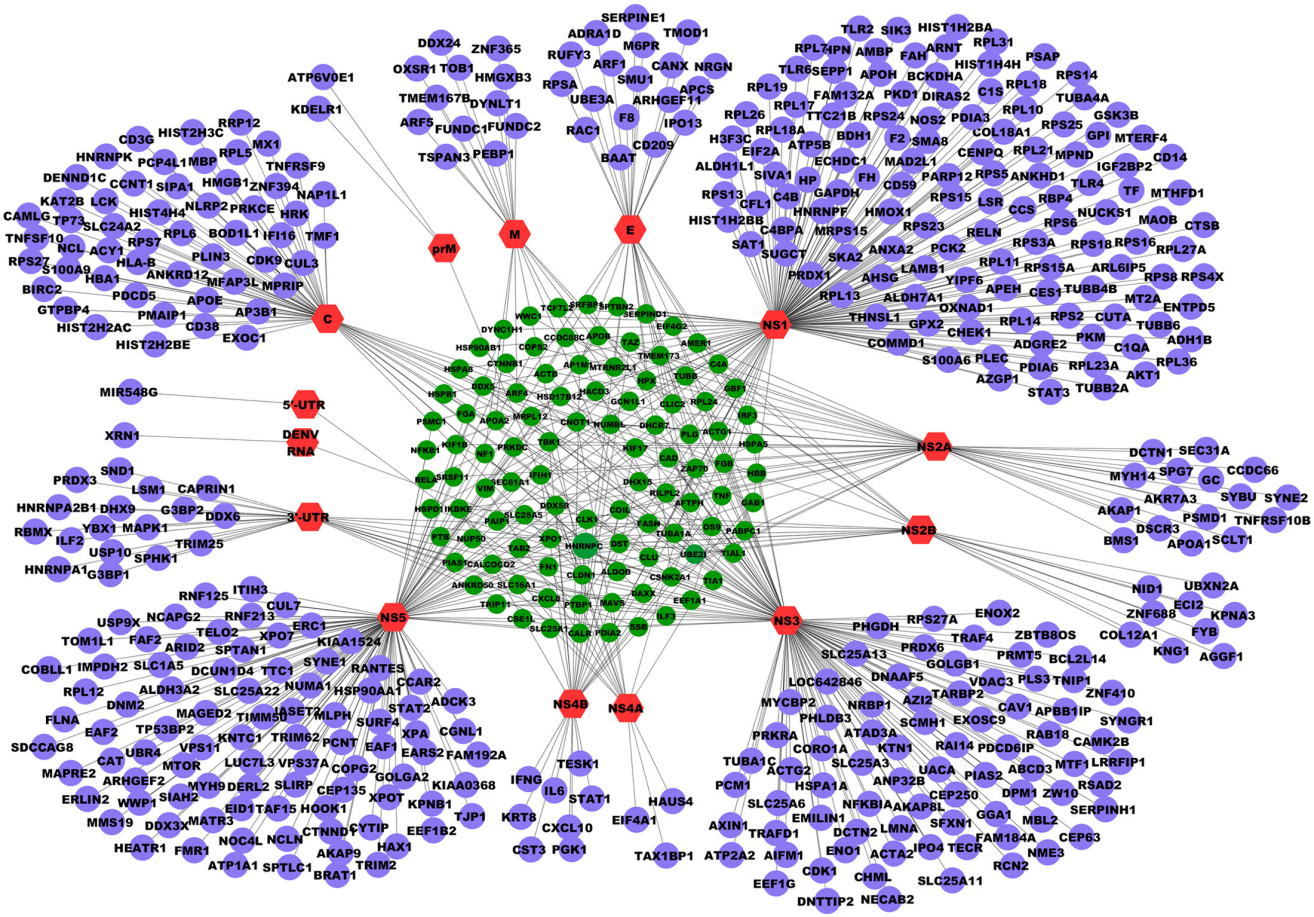
Visualization of the network of direct dengue-human interactions. The network of direct physical interactions of dengue–human components was visualized using Cytoscape. Pink hexagons represent dengue viral components, blue circles represent human proteins that interact with one dengue component and green circles represent human proteins that interact with more than one dengue protein.

Most of the direct interactions reported in literature have been determined using affinity purification, pull down assays, co-localization by microscopic techniques or high-throughput techniques, such as yeast two-hybrid (Y2H) and tandem affinity purification followed by mass spectrometry. Literature describing interactions between viral and human proteins from articles that have been determined through any of the above experiments were extracted and manually cataloged (S2 Dataset). There have been few studies that employ computational methods which have used protein structural similarity to predict PPIs of dengue viral proteins in mosquitoes and human hosts. Predicted interactions from such papers were extracted only if there was prior evidence of the human protein to be important in dengue viral infection. The network of direct interactions between dengue viral components and human proteins was visualized in Cytoscape (Fig 1).

There are few human proteins that can interact with more than one viral protein. Table 2 numerically summarizes the human proteins that interact with different dengue viral components (both viral proteins and RNA). Consolidated list of human proteins that can interact with 3 and more viral components is given in S3 Dataset. Two human proteins UBE2I and CSNK2A1 interact with multiple viral components, but most of their interactions are predicted interactions. Two proteins, IKKε and PTBP1, are shown to directly interact with 5 different viral components. IKKε interacts with non-structural (NS) proteins 2A, 2B, 3, 4A and 4B and these associations play an important role in inhibiting the interferon signaling pathway [22, 23]. IKKε is also known to interact with proteins of other viruses such as measles [24], vaccinia [25], arenavirus[26], hepatitis B [27] and hepatitis C [28]. PTBP1 is an integral part of the viral replication complex and interacts with both the 5’- and 3’- UTR of dengue viral RNA, and modulates negative strand RNA synthesis [29, 30], as it does for many other viruses such as coxsackie [31], Japanese encephalitis [32] and hepatitis C [33].

**Table 2:** The number of human proteins involved in direct interactions with N number of dengue viral proteins. The number of human proteins that can interact with N = 1–7 viral components along with the percentages they constitute are shown in the table.

S.No.	Number of human proteins	Number of DENV proteins (N)	Percent of total direct interactions identified
1	432	1	80.7
2	77	2	14.4
3	16	3	3
4	5	4	0.9
5	3	5	0.6
6	1	6	0.2
7	1	7	0.2

#### Indirect or functional interactions

The most commonly used experimental approach to elucidate the function of a gene, in this case the role of a human protein in viral replication or pathogenesis, is the selective deletion of its expression or activity by several approaches including mutational studies, gene silencing and inhibition studies. There have been many studies that use any one or more of the above mentioned approaches to determine human proteins that play an important role in viral replication called the dengue virus host dependency factors (DVHFs). Apart from the above described methods, there have been studies carried out using patient’s blood or serum in order to determine biomarkers in dengue infection. Data from such studies were extracted as indirect interactions (S2 dataset). Among the 382 indirect interactions identified, 23% of the interactions are reported in more than one publication.

#### Differential expressed interactions

With the development of high-throughput transcriptomic and proteomic technologies such as microarrays, MALDI and mass spectrometry, it has become possible to identify genes that are differentially expressed in dengue infected cell lines and patient whole blood, serum and PBMCs. The differentially expressed proteins obtained using such approaches provide valuable insights into the dynamic relationships between the host cell and the virus during the course of infection. Majority of the interactions in DenHunt are differentially expressed interactions. Out of the 4120 differentially expressed genes during dengue infection, 222 genes are consistently up or down regulated in at least 4 different publications (S2 Dataset).

### Pathway enrichment analysis

**Fig 2:**
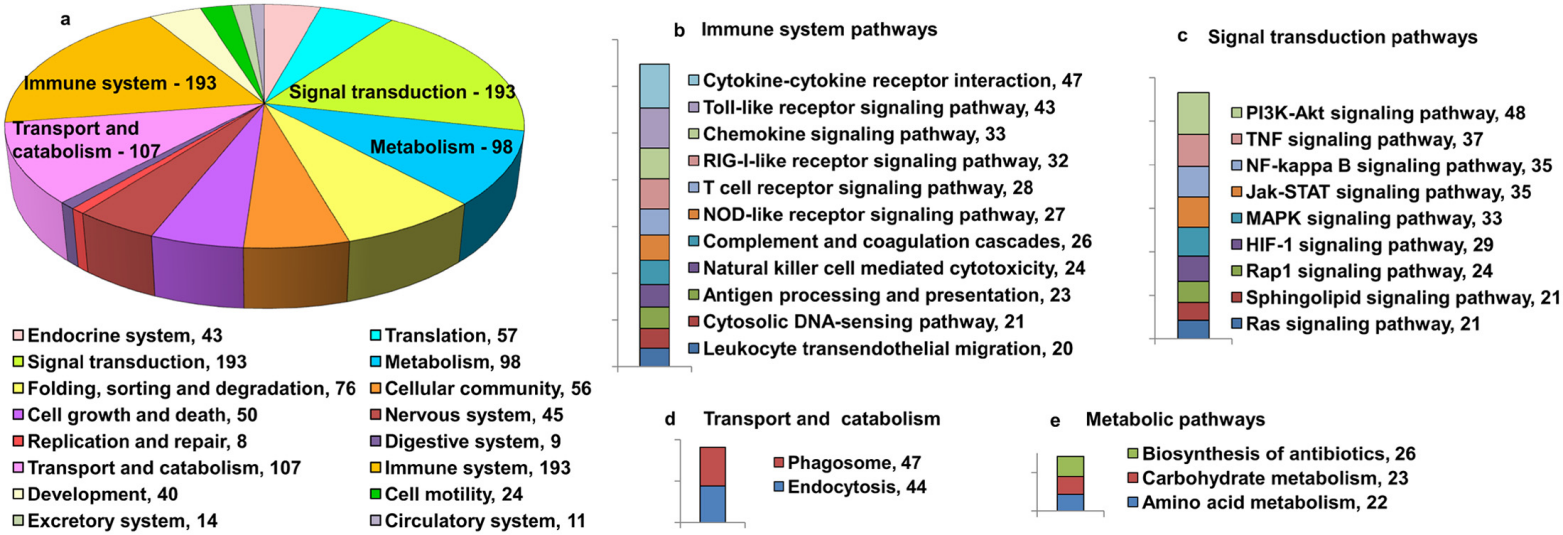
Enrichment of KEGG pathways for the dengue virus interacting human proteins. Pathway enrichment analysis for dengue virus interacting proteins was carried out using WebGestalt. Only pathways that have 3 and more dengue virus interacting proteins and an adj p-value of ≤ 0.01 are selected. The KEGG pathways that were enriched were grouped into broad categories as mentioned in the KEGG pathway database. (a) The broad categories and their total number of dengue virus interacting human proteins are plotted as a pie chart. The color code for each category is given below the pie chart. Pathways that have 20 and more dengue virus interacting proteins belonging to the top 4 broad categories are plotted as bar graph where (b) represents immune system pathways, (c) represents signal transduction pathways, (d) represents transport and catabolism and (e) represents metabolic pathways. In all cases numbers indicates the number of genes identified in each pathway.

It is widely accepted that successful invasion of the host by the pathogen involves targeting multiple components of host cellular machinery. To better understand the molecular mechanisms underlying dengue pathogenesis, we determined statistically significant over-represented or enriched KEGG pathways in the dengue virus interacting human proteins. A comprehensive list of human proteins which are involved in direct interactions, indirect interactions and are reported to be DEGs in at least 4 different publications in dengue infected cell lines or patients (S1 Dataset) was subjected to gene set enrichment analyses using the online tool WebGestalt. The reason we selected genes or proteins that were DEGs in at least 4 publications was to pick a high confidence gene list for all our downstream analysis as the techniques used to determine DEGs, such as microarray and proteomics, are greatly prone to errors. KEGG pathways that contained at least 3 dengue interacting proteins and had an adjusted p-value ≤ 0.01 were selected. We obtained 158 KEGG pathways of which 87 belonged to normal biological processes and 71 to disease pathways. To ease representation of these enriched pathways, the pathways belonging to normal biological processes were grouped into broad categories as described at the KEGG database (S4 Dataset) and plotted as a pie chart (Fig 2a). The top 4 broad categories, which have the maximum number of representative genes, are Signal transduction (193 genes), Immune system (193 genes), Transport and catabolism (107 genes) and Metabolism (98 genes). Pathways that have 20 and more dengue interacting human proteins that belong to these top 4 broad categories are represented in Fig 2b, 2c, 2d and 2e.

Most of the host proteins identified as dengue virus receptors play important roles in cellular signaling, pathogen recognition and innate immune response [34]. Since dengue virus uses multiple receptors to enter cells, it is likely that viral infection will activate several cellular signaling and immune pathways. As expected, many proteins involved in cellular signaling and immune system pathways interact directly or indirectly with dengue virus as can be seen in Fig 2a, 2b and 2c. Apart from these pathways, dengue virus replication is closely associated with processes that are involved in transport and catabolism such as endocytosis and phagocytosis. The vesicular trafficking processes, such as receptor mediated endocytosis and the classical secretory exocytosis, are intimately associated with processes of viral entry, maturation and exit [35, 36]. Therefore, it is necessary for the proteins involved in the vesicular trafficking system to interact with viral proteins extensively. Corroborating these studies, we identified many proteins belonging to endocytosis and phagocytosis pathways interacting with DENV (Fig 2d). We also observe quite a few dengue virus interacting human proteins belonging to the amino acid and carbohydrate metabolism processes (Fig 2e). This is expected, as viral replication relies heavily on the host metabolic resources (amino acids and nucleotides) to produce large numbers of progeny which constitute the viral RNA and proteins.

**Fig 3:**
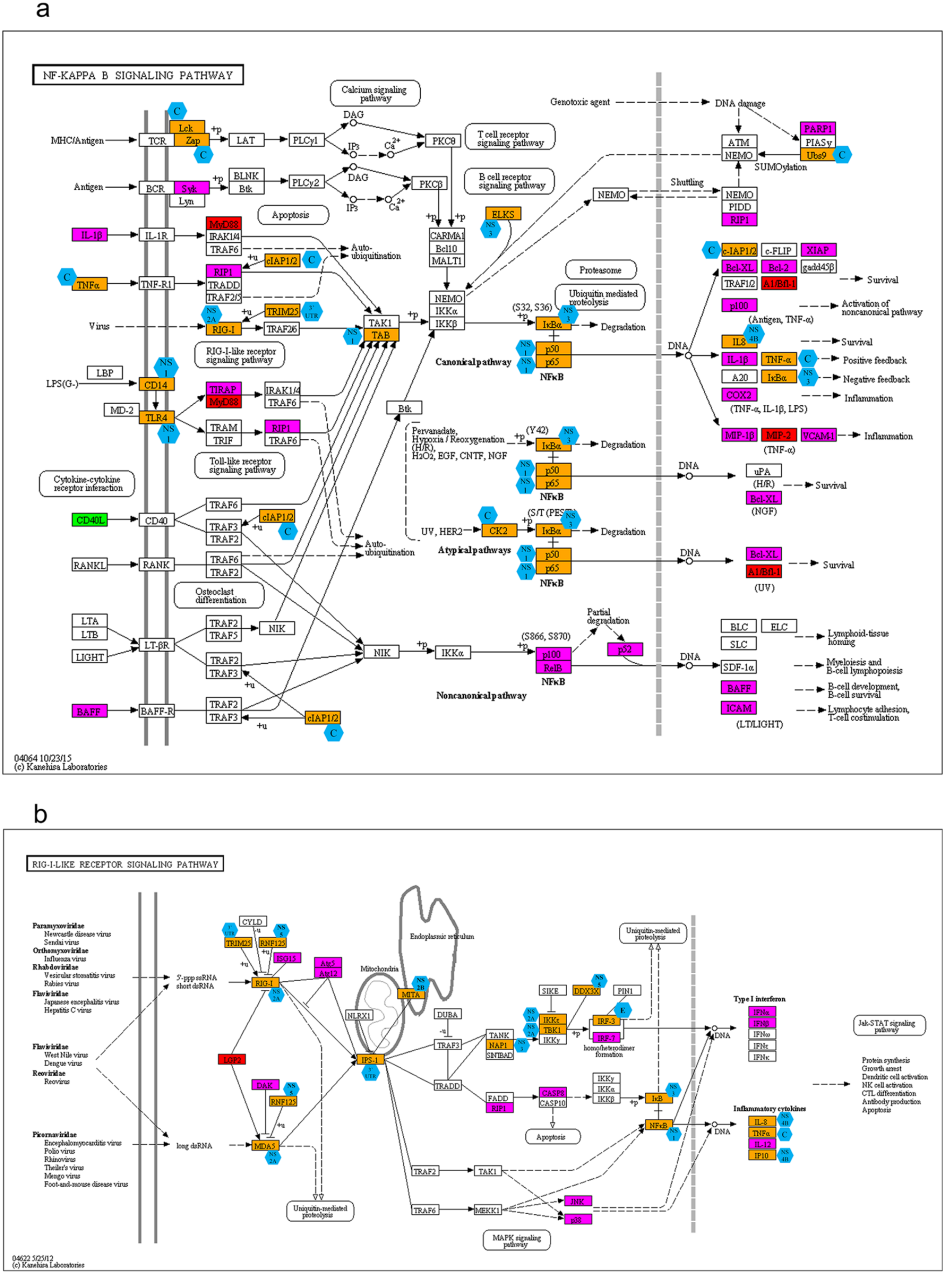
Representation of dengue viral interacting proteins in NF-κB and RIG-I-like receptor signaling pathway. The dengue virus interacting proteins are mapped to the KEGG pathways using the “Search&Colour Pathway” tool from the KEGG database. The proteins that are involved in direct interactions are colored orange, indirect interactions are colored in pink, up-regulated proteins are colored red and down-regulated proteins are colored green. The viral components are depicted as blue hexagons. Fig 3a represents NF-κB and 3b RIG-I-like receptor signaling pathway.

Pathway enrichment analysis showed that majority of the dengue virus interacting human proteins belongs to the signal transduction and immune system pathways. Further, we mapped the dengue virus interacting human proteins to the KEGG pathway maps using the “Search&Colour Pathway” tool from the KEGG database. All the graphical images for the pathways, which have 20 and more dengue interacting human proteins, belonging to signal transduction and immune systems categories are given in the S1 Fig. Here, we describe the role of two pathways, NF-κB and retinoic acid-inducible gene I (RIG-I)-like receptor signaling pathway, in viral infection (Fig 3). These pathways play an important role in viral infection in general and in particular in dengue infection as evidenced by at least 85 papers that analyze proteins belonging to these pathways in connection to dengue viral infection.

NF-κB is termed the central mediator of the immune response and is an attractive target for many pathogens including dengue virus [37]. Dengue viral protease 2B-3 interacts with and cleaves IκBα/β, a NF-κB inhibitor, and activates NF-κB [38]. Many of the activators of NF- κB that help in degradation of IκBα/β, also interact with dengue viral proteins (Fig 3a). The other immune system pathway discussed here is the RIG-I-like receptor signaling pathway which responds to viral infection by recognizing viral replication intermediates and double stranded RNA (dsRNA) and activate interferon regulatory factors (IRFs). They, then, turn on transcription of interferons alpha and beta, as well as other interferon-induced genes [39, 40]. As can be seen from Fig 3b, different viral proteins interact with many proteins of the RIG signaling pathway to inhibit IRF3 and thereby hamper IFNα/β production [23].

### Association of dengue interacting proteins with infectious and noninfectious diseases

**Fig 4:**
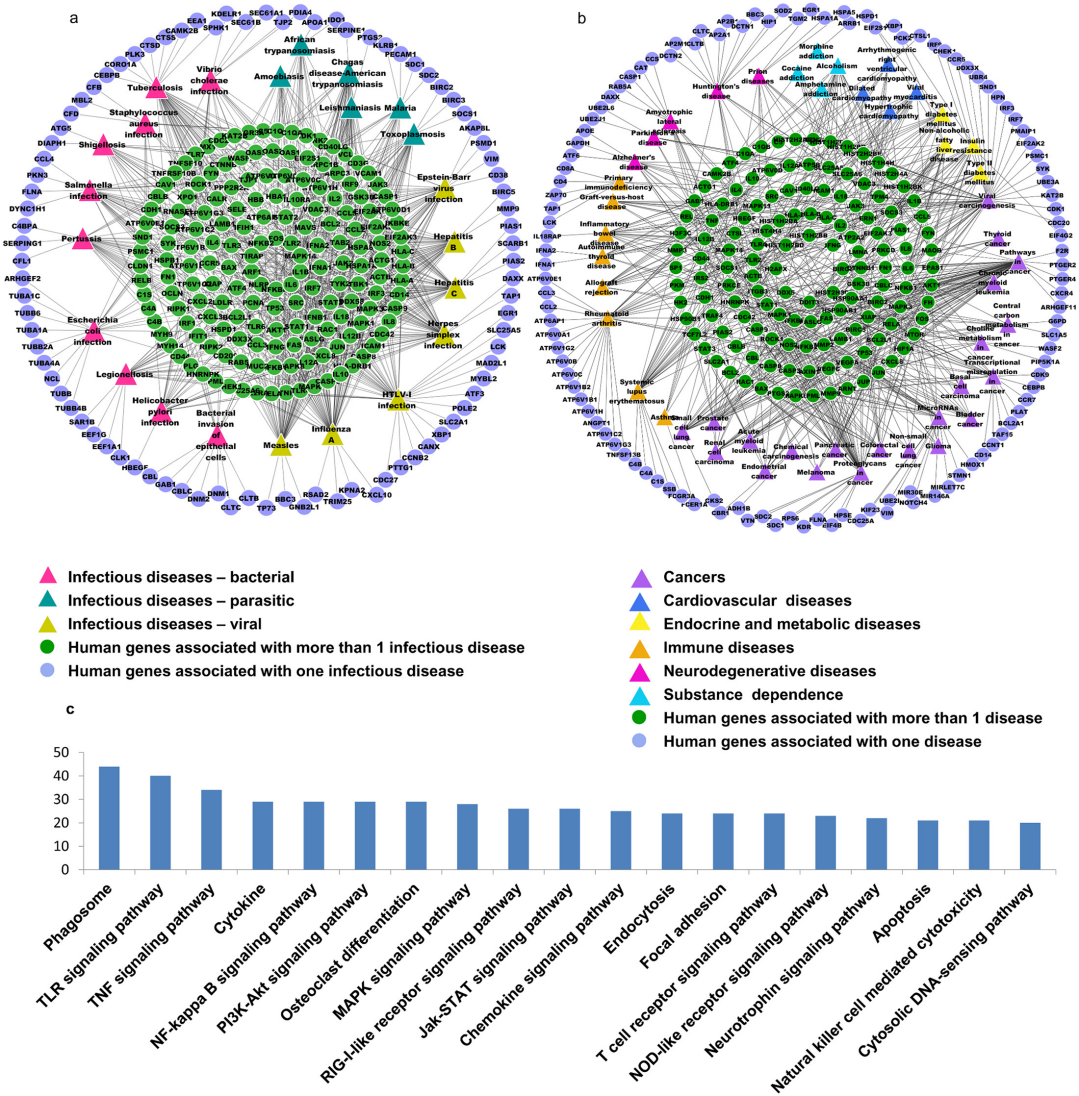
Association of dengue viral interacting human proteins with infectious diseases and non-infectious diseases. The dengue virus interacting human proteins and their associated a) infectious and b) non-infectious diseases are visualized as a network using Cytoscape. The diseases are represented as triangles and are color coded based on the disease group they belong to. The genes that are associated with only in one disease are represented as violet colored circles and those that are associated with more than one disease are represented as green colored circles. c) Pathway analysis of the dengue virus interacting proteins that are associated with both infectious and non-infectious diseases. KEGG pathways that had 20 or more dengue virus proteins associated with both infectious and non-infectious diseases using KEGG Mapper—Search Pathway is shown as a bar graph.

The manifestation and severity of an infectious disease depends on the ability of a pathogen to interfere with host cell functions and defense. Indeed, during pathogen-host co-evolution, hosts have developed an armory of complex defense mechanisms to eliminate the pathogens. Conversely, pathogens have evolved strategies, in part driven by molecular interactions, to evade host cellular defense and to sustain their control over the cellular machinery. We studied the involvement of the dengue viral interacting human proteins in other infectious diseases that belong to the bacterial, viral and parasitic category as given in the KEGG database using WebGestalt (S5 Dataset). The dengue virus interacting human proteins and their association with other infectious diseases was visualized in Cytoscape (Fig 4a). 273 dengue interacting human proteins are associated with other infectious diseases and 168 of them are associated with more than one infectious disease. A similar result was observed in a study where they analyzed the landscape of the human proteins that interact with pathogens and showed that proteins of many viruses share the same interacting human protein, and thus share common infection and immune evasion strategies [41].

There have been studies which suggest that the molecular perturbations triggered by virus-host PPIs could also be involved in many non-infectious diseases of genetic or environmental predisposition [42]. We explored the participation of dengue viral interacting human proteins in the pathologies of various complex non-infectious diseases caused by genetic or environmental factors as given in KEGG database using the tool WebGestalt (S6 Dataset). The dengue viral interacting genes and their association with other complex non-infectious diseases was visualized in Cytoscape (Fig 4b). 249 dengue interacting human proteins are associated with non-infectious diseases and 135 of them are associated with more than one non-infectious disease. The analysis shows that dengue virus interacting proteins are also involved in a wide range of pathologies, with most of them related to cancers followed by auto immune disorders.

We also observed 140 dengue interacting human proteins associated with both infectious and non-infectious diseases (S7 Dataset). This shows that many human proteins of dengue-human interactome are also involved in the response to pathogenic infections and other complex non-infectious diseases. Pathway analysis of the common genes, using KEGG Mapper, showed an enrichment of pathways belonging to immune system and signal transduction pathways (Fig 4c). This implies an unexpected overlapping between the pathways associated with infectious and non-infectious diseases and a perturbation in these pathways might result in the manifestation of diseases.

### Dengue-Human interactome, a potential resource for drug repurposing

Drug discovery for dengue viral diseases poses unique challenges on many fronts. One major drawback in the development of drugs or vaccines against dengue is that the disease is caused by four DENV serotypes, with all of them co-circulating in many parts of the world. An effective vaccine or drug must act on all 4 serotypes. The other problem is the unavailability of reliable animal models for credible preclinical evaluation of *in vivo* efficacy of investigational drugs and vaccines. Analysis of the literature and the patent databases shows that several strategies are being used to develop anti-virals against dengue diseases. Drugs in the pipeline are targeted against (a) viral factors which include viral protein inhibitors (NS3pro inhibitors, NS3 helicase inhibitors, RdRp inhibitors, a-glucosidase inhibitors) [43–50], (b) host cell entry inhibitors [51–56], (c) host factors that are involved in the host immune response [57, 58] and (d) unknown targets which include small molecule inhibitors [59, 60] and herbal inhibitors [61–64].

Majority of the drugs, mentioned above, are targeted towards viral proteins which could force the virus to evolve into resistant strains for survival. Therefore, therapeutics which target host proteins required by pathogens to replicate and persist within the host organism could be an attractive alternative. As seen in the previous section, many human proteins of the dengue-human interactome are involved in the pathogenesis of other infectious and non-infectious diseases and thus represent a powerful resource to identify broad-spectrum drugs. Further, the possibility to target dengue virus-human protein interactions considerably broadens the landscape of drugs that could be developed against dengue infections.

Drug repurposing or repositioning is the process of finding new indications for existing drugs [65]. The advantages of this approach are lower costs of drug development [65, 66]. A number of success stories, such as sildenafil (Viagra) repositioned from a common hypertension drug to a therapy for erectile dysfunction [66] and thalidomide repositioned to treat multiple myeloma and leprosy complications [67], support these approaches. We mined potential FDA drugs against dengue virus interacting human proteins, used to treat other diseases.

In order to extract potential drug targets from the list of dengue virus interacting human proteins, we pulled out only those proteins which were important for viral replication that we term as potential DVHFs. 263 dengue virus interacting proteins were considered to be potential DVHFs because they were described to be essential for viral replication in publications, as inhibiting these proteins led to a reduction in viral replication (S8 Dataset). We, then, extracted the FDA approved drugs that had known pharmacological action against the DVHFs from DrugBank (Table 3, S9 Dataset). 20 of the 263 DVHFs had known FDA approved drugs targeted against them and 9 of these DVHFs were associated with more than one infectious or non-infectious disease. Two of the proteins CCR5 and HMGCR are already established drug targets against another virus, HIV [68, 69]. Maraviroc and lovastatin, inhibitors of CCR5 and HMGCR respectively, have already been shown to inhibit dengue viral replication in *in vitro* studies [70, 71].These drugs could be tested for their anti-dengue viral effect and it is possible that some of these drugs, either singly or in synergistic combinations may prove to be effective antiviral agents. The use of such drug repositioning strategies which makes the use of known targets, drugs and disease pathways would lead to faster computer to bench studies and reduce the risk and cost of drug discovery approaches to Neglected Tropical Diseases such as diseases caused by dengue virus.

**Table 3:** List of the DVHFs and the number of FDA drugs, infectious diseases and non-infectious diseases associated with them. This table contains a list of DVHFs, the number FDA approved drugs against the DVHFs which are used to treat other diseases and the number of infectious and non-infectious diseases associated with the DVHFs.

Gene symbol	Gene id	No of FDA drugs	Number of Non-infectious diseases associated with the protein	Number of Infectious diseases associated with the protein
ATP6V1B2	526	1	1	2
AVPR1B	553	1	-	-
CAMLG	819	1	-	-
CCR5	1234	1	1	1
FASN	2194	1	-	-
GANAB	23193	1	-	-
IL6	3569	1	9	13
JUN	3725	1	10	10
M6PR	4074	1	-	-
MAPK11	5600	1	2	11
NPR2	4882	1	-	-
PLIN3	10226	1	-	-
SERPINE1	5054	1	-	1
SRC	6714	1	3	5
IL1B	3553	2	7	12
DRD4	1815	10	-	-
HMGCR	3156	10	-	-
TNF	7124	12	15	14
CHRNA2	1135	26	-	-
HTR2A	3356	54	-	-

## Discussion

The underlying aim of all dengue viral research is to understand the pathogenesis associated with dengue infection and the development of effective clinical interventions to inhibit viral infection and replication, thereby preventing progression towards to the severe forms of the disease such as DHF and DSS. Dengue–host interaction data, a representation of existing knowledge about dengue infection on a molecular level, will thus be invaluable to future research in this area. DenHunt was developed as a user friendly public repository to capture and organize manually curated information from the available scientific literature on the interactions between dengue virus and host proteins

This information will be of immense importance in improving our understanding of how the dengue virus replicates in the context of the whole cell and how the host-viral interactions control dengue replication and mediate viral pathogenesis. Such insights can also be extrapolated to understand mechanisms of infectious diseases in general. DenHunt could be used to make detailed maps tracking cellular interactions that drive dengue viral replication, and provides a discovery space to the research community for researching and better understanding the dengue viral pathogenesis. An example of how such databases can aid in understanding viral replication is provided by Brass et al. [72] who used the information in the HHPID database to systematically analyze and categorize human proteins required for HIV-1 replication.

Our pathway analysis section shows how dengue virus targets multiple components of the same pathway to mediate effects such as apoptosis or inhibition of IFNα/β production. Although the approach adopted here is purely qualitative, we have amply demonstrated how an integrated repository such as DenHunt could be used to harness already existing data to elucidate dengue viral pathogenesis mechanisms. The key to gain new understanding from DenHunt in viral pathogenesis would lie in its integration with other sources of multidimensional data such as time-course dengue infected gene expression data in the context of an integrated dengue–host PPIs to identify “activated modules” or “highest activity paths” as has been done for other diseases such as HIV [73] and *Mycobacterium tuberculosis* [74]. Therefore, these networks could serve as an initiating point for various systems level modeling and computational studies.

The high mutation rate and development of resistance strains of RNA viruses can quickly restrain the effectiveness of drugs targeting viral proteins. This observation has led to research on developing drugs that disrupt virus-host interactions rather than viral proteins itself. Few such successful examples with regard to HIV are the viral entry inhibitor maraviroc [68], and a fusion inhibitor enfuvirtide [75]. The availability of a large set of dengue interacting human proteins raises the possibility of identifying putative novel drug targets by *in silico* methods. We have illustrated that at least seven percent of the dengue interacting human proteins are associated with other infectious and noninfectious diseases and a subset of these proteins are already reported to be targeted by FDA-approved drugs to treat other diseases.

### Conclusions

We have developed a comprehensive dengue virus-human interaction network database called DenHunt, which contains a compilation of a curated set of experimentally verified dengue-human interactions. Our database would enable construction and visualization of the essential map of the dynamic networks of interactions that occur during viral life cycle in humans. Detailed characterization of the relationships between these interactions that include multidimensional data given in the database such as direct physical interactions, indirect interactions, gene expression patterns, gene silencing studies, virus serotype, cell type, disease stage, etc., will lead to improved understanding of the conflict between dengue and its human host.

## Supporting Information

S1 Table The keywords used in the queries. This list contains the keywords used in queries along with dengue viral proteins in PubMed to extract articles that contain information of dengue—human interactions.
(PDF)

S2 Table List of human and viral genes mentioned in the paper. Table A has the list of human genes mentioned in the paper, their official gene symbol and Entrez gene id. Table B has the list of viral genes mentioned in the paper, their official gene symbol and Reference Protein accession id.
(PDF)

S1 Fig Representation of dengue viral interacting protein belonging to various signal transduction and immune system pathway. Representation of dengue viral interacting proteins in (a) PI3K-Akt signaling pathway, (b) TNF signaling pathway, (c) NF-kappa B signaling pathway, (d) Jak-STAT signaling pathway, (e) MAPK signaling pathway, (f) HIF-1 signaling pathway, (g) Rap1 signaling pathway, (h) Sphingolipid signaling pathway, (i) Ras signaling pathway, (j) Cytokine-cytokine receptor interaction, (k) Toll-like receptor signaling pathway, (l) Chemokine signaling pathway, (m) RIG-I-like receptor signaling pathway, (n)T cell receptor signaling pathway, (o) NOD-like receptor signaling pathway, (p) Complement and coagulation cascades, (q) Natural killer cell mediated cytotoxicity, (r) Antigen processing and presentation, (s) Cytosolic DNA-sensing pathway and (t) Leukocyte transendothelial migration. The dengue virus interacting proteins are mapped to the pathways using the “Search&Colour Pathway” tool from the KEGG database. The proteins that are involved in direct interactions are colored orange, indirect interactions are colored in pink, up-regulated proteins are colored red and down-regulated proteins are colored green. The viral components are depicted as blue hexagons.
(PDF)

S1 Dataset List of dengue viral interacting human proteins used for pathway analysis. List of dengue virus interacting human genes that are identified to: i) interact directly with viral proteins, ii) indirectly affect dengue infection and iii) are reported to be DEGs (consistently up or down regulated in dengue infection) in at least 4 different publications in dengue infected cell lines or patients. This list is used for subsequent pathway and disease analyses.
(XLSX)

S2 Dataset The consolidated list of direct, indirect and differentially expressed dengue virus interacting genes. This excel file contains the manually curated dengue virus-human interactions that belong to the 1) direct physical interactions, 2) indirect interactions and 3) differentially expressed genes.
(XLSX)

S3 Dataset Human proteins that interact with 3 and more viral components. All the human proteins that physically interact with 3 and more viral components are listed in this table.
(XLSX)

S4 Dataset List of enriched KEGG pathways involved in normal biological processes of the dengue virus interacting human proteins. The dengue virus interacting proteins are subjected to pathway enrichment using WebGestalt. Only pathways that have 3 and more dengue virus interacting proteins and an adj p-value of ≤ 0.01 are selected. This is the list of enriched pathways of the dengue virus interacting proteins belonging to normal biological processes.
(XLSX)

S5 Dataset Enriched infectious disease KEGG pathways associated with dengue virus interacting proteins. The infectious diseases of the bacterial, viral and parasitic category enriched in dengue virus interacting proteins using WebGestalt. Only pathways that have 3 and more dengue virus interacting proteins and an adj p-value of ≤ 0.01 are selected.
(XLSX)

S6 Dataset Enriched non-infectious disease KEGG pathways associated with dengue virus interacting genes. List of statistically significant non infectious disease KEGG pathways, which are caused by environmental and genetic factors, associated with dengue virus interacting proteins identified using Webgestalt. Only pathways that have 3 and more dengue virus interacting proteins and an adj p-value of ≤ 0.01 are selected.
(XLSX)

S7 Dataset Dengue virus interacting human proteins involved in both infectious and non-infectious diseases. This file contains the list of dengue virus interacting human proteins that are also involved in infectious and non-infectious diseases.
(XLSX)

S8 Dataset Dengue virus interacting human proteins that are considered to be DVHFs. This file contains the list of dengue virus interacting human proteins that have been found to be essential for dengue viral replication as inhibiting these proteins caused a reduction in viral replication.
(XLSX)

S9 Dataset List of DVHFs and their FDA approved drugs. This file contains the list of dengue virus interacting human proteins that have been found to be essential for dengue viral replication along with known FDA approved drugs against them.
(XLSX)

S1 Checklist PRISMA checklist of items for the meta analysis carried out to construct the database DenHunt. (DOC)

S1 Flowchart PRISMA Flow chart of the curation process to construct DenHunt. (DOC)
