# Supplementary material for: DenHunt - A Comprehensive Database of the Intricate Network of Dengue-Human Interactions
Source: PLoS Negl Trop Dis. 2016 Sep 12;10(9):e0004965. doi: 10.1371/journal.pntd.0004965 (PMC5019383; doi:10.1371/journal.pntd.0004965)
Supplement: S1 Table — This list contains the keywords used in queries along with dengue viral proteins in PubMed to extract articles that contain information of dengue—human interactions. (PDF) [file pntd.0004965.s001.pdf]

S1 Table: The keywords used in the queries. This list contains the keywords used in queries along with dengue viral proteins in PubMed to extract articles that contain information of dengue - human interactions.

Keywords used in search			
activated	degrades	inactivates	processed
associates	dephosphorylated	incorporates	recruited
binds	depolymerizes	induces	regulated
cleaves	destabilized	inhibited	relocalized
colocalizes	disrupted	interacts	requires
competes	downregulated	mediated	rescued
complexes	enhanced	modified	sensitizes
cooperates	exported	modulated	stabilized
phosphorylated	imported	over expressed	stimulated
synergizes	ubiquitinated	upregulated	sumoylated
