# Supplementary material for: DenHunt - A Comprehensive Database of the Intricate Network of Dengue-Human Interactions
Source: PLoS Negl Trop Dis. 2016 Sep 12;10(9):e0004965. doi: 10.1371/journal.pntd.0004965 (PMC5019383; doi:10.1371/journal.pntd.0004965)
Supplement: S2 Table — Table A has the list of human genes mentioned in the paper, their official gene symbol and Entrez gene id. Table B has the list of viral genes mentioned in the paper, their official gene symbol and Reference Protein accession id. (PDF) [file pntd.0004965.s002.pdf]

S2 Table: List of human and viral genes mentioned in the paper. Table A has the list of human genes mentioned in the paper, their official gene symbol and Entrez gene id. Table B has the list of viral genes mentioned in the paper, their official gene symbol and Reference Protein accession id.

**S2 Table A**		
**Gene Symbols of human proteins mentioned in the paper**	**Official Gene Symbol**	**Entrez Gene id**
UBE2I	UBE2I	7329
CSNK2A1	CSNK2A1	1457
IKKε	IKBKE	9641
PTBP1	PTBP1	5725
NF-κB	NFKB1	4790
NF-κB	RELA	5970
IκBα/β	NFKBIA	4792
IκBα/β	NFKBIB	4793
IRF3	IRF3	3661
IFNα/β	IFNA1	3439
IFNα/β	IFNB1	3456
CCR5	CCR5	1234
HMGCR	HMGCR	3156

**S2 Table B**		
**Gene Symbol of dengue viral proteins mentioned in the paper**	**Official Gene Symbol**	**GenBank Reference Sequence Protein id**
Dengue viral protein NS2A	NS2A	NP_739585.2
Dengue viral protein N2B	NS2B	NP_739586.2
Dengue viral protein NS3	NS3	NP_739587.2
Dengue viral protein NS4A	NS4A	NP_739588.2
Dengue viral protein NS4B	NS4B	NP_739589.2
