# Supplementary material for: DenHunt - A Comprehensive Database of the Intricate Network of Dengue-Human Interactions
Source: PLoS Negl Trop Dis. 2016 Sep 12;10(9):e0004965. doi: 10.1371/journal.pntd.0004965 (PMC5019383; doi:10.1371/journal.pntd.0004965)
Supplement: S1 Fig — Representation of dengue viral interacting proteins in (a) PI3K-Akt signaling pathway, (b) TNF signaling pathway, (c) NF-kappa B signaling pathway, (d) Jak-STAT signaling pathway, (e) MAPK signaling pathway, (f) HIF-1 signaling pathway, (g) Rap1 signaling pathway, (h) Sphingolipid signaling pathway, (i) Ras signaling pathway, (j) Cytokine-cytokine receptor interaction, (k) Toll-like receptor signaling pathway, (l) Chemokine signaling pathway, (m) RIG-I-like receptor signaling pathway, (n)T cell receptor signaling pathway, (o) NOD-like receptor signaling pathway, (p) Complement and coagulation cascades, (q) Natural killer cell mediated cytotoxicity, (r) Antigen processing and presentation, (s) Cytosolic DNA-sensing pathway and (t) Leukocyte transendothelial migration. The dengue virus interacting proteins are mapped to the pathways using the “Search&Colour Pathway” tool from the KEGG database. The proteins that are involved in direct interactions are colored orange, indirect interactions are colored in pink, up-regulated proteins are colored red and down-regulated proteins are colored green. The viral components are depicted as blue hexagons. (PDF) [file pntd.0004965.s003.pdf]

S1 Fig: Representation of dengue viral interacting protein in signal transduction and immune system pathway. Representation of dengue viral interacting proteins in (a) PI3K-Akt signaling pathway, (b) TNF signaling pathway, (c) NF-kappa B signaling pathway, (d) Jak-STAT signaling pathway, (e) MAPK signaling pathway, (f) HIF-1 signaling pathway, (g) Rap1 signaling pathway, (h) Sphingolipid signaling pathway, (i) Ras signaling pathway, (j) Cytokine-cytokine receptor interaction, (k) Toll-like receptor signaling pathway, (l) Chemokine signaling pathway, (m) RIG-I-like receptor signaling pathway, (n) T cell receptor signaling pathway, (o) NOD-like receptor signaling pathway, (p) Complement and coagulation cascades, (q) Natural killer cell mediated cytotoxicity, (r) Antigen processing and presentation, (s) Cytosolic DNA-sensing pathway and (t) Leukocyte transendothelial migration. The dengue virus interacting proteins are mapped to the pathways using the “Search&Colour Pathway” tool from the KEGG database. The proteins that are involved in direct interactions are colored orange, indirect interactions are colored in pink, up-regulated proteins are colored red and down-regulated proteins are colored green. The viral components are depicted as blue hexagons.

a

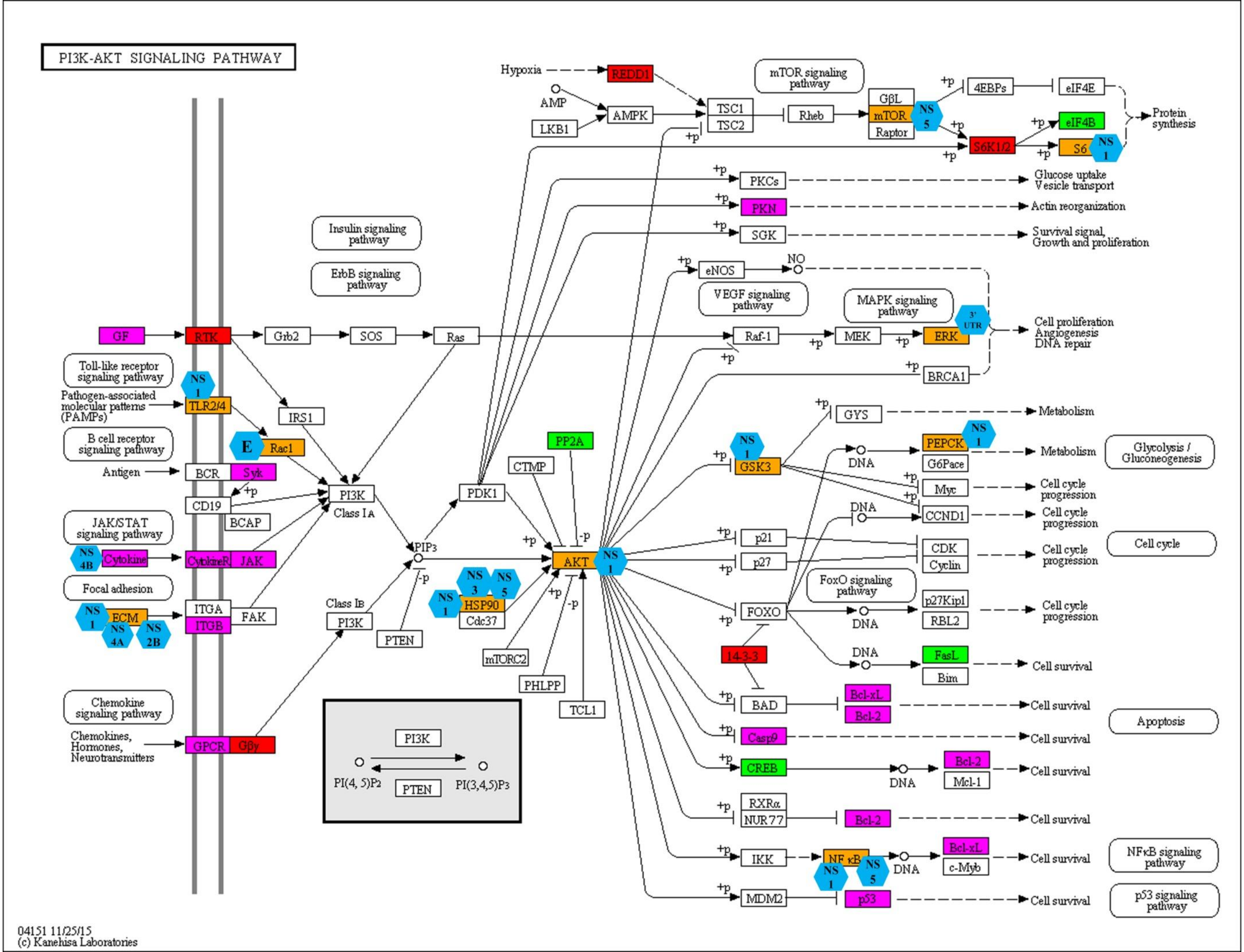
PI3K-AKT SIGNALING PATHWAY
Hypoxia
REDD1
AMP
AMPK
LKB1
TSC1
TSC2
Rheb
mTOR signaling pathway
GβL
mTOR
Raptor
NS 5
+p
4EBPs
eIF4E
eIF4B
S6K1/2
S6
NS 1
Protein synthesis
PKCs
Glucose uptake
Vesicle transport
PKN
Actin reorganization
SGK
Survival signal, Growth and proliferation
Insulin signaling pathway
ErbB signaling pathway
eNOS
NO
VEGF signaling pathway
MAPK signaling pathway
3' UTR
GF
RTK
Grb2
SOS
Ras
Raf-1
MEK
ERK
Cell proliferation
Angiogenesis
DNA repair
BRCA1
Toll-like receptor signaling pathway
Pathogen-associated molecular patterns (PAMPs)
TLR2/4
IRS1
GYS
Metabolism
B cell receptor signaling pathway
E
Rac1
PP2A
PEPCK
G6Pace
Glycolysis / Gluconeogenesis
Antigen
BCR
Syk
GSK3
CTMP
DNA
Myc
Cell cycle progression
CD19
BCAP
PI3K
Class IA
PDK1
CCND1
JAK/STAT signaling pathway
NS 4B
Cytokine
CytokineR
JAK
PIP3
AKT
-p
p21
p27
CDK
Cyclin
Cell cycle
Focal adhesion
NS 3
HSP90
Cdc37
FoxO signaling pathway
p27Kip1
RBL2
ECM
NS 4A
NS 2B
ITGA
ITGB
FAK
Class IB
PTEN
mTORC2
FOXO
14-3-3
FasL
Bim
Cell survival
PHLPP
TCL1
BAD
Bcl-xL
Bcl-2
Apoptosis
Chemokine signaling pathway
Chemokines, Hormones, Neurotransmitters
GPCR
Gβγ
PI(4, 5)P2
PI(3,4,5)P3
Casp9
CREB
Mcl-1
RXRα
NUR77
IKK
NF-κB
c-Myb
NFκB signaling pathway
MDM2
p53
p53 signaling pathway
04151 11/25/15
(c) Kanehisa Laboratories

b

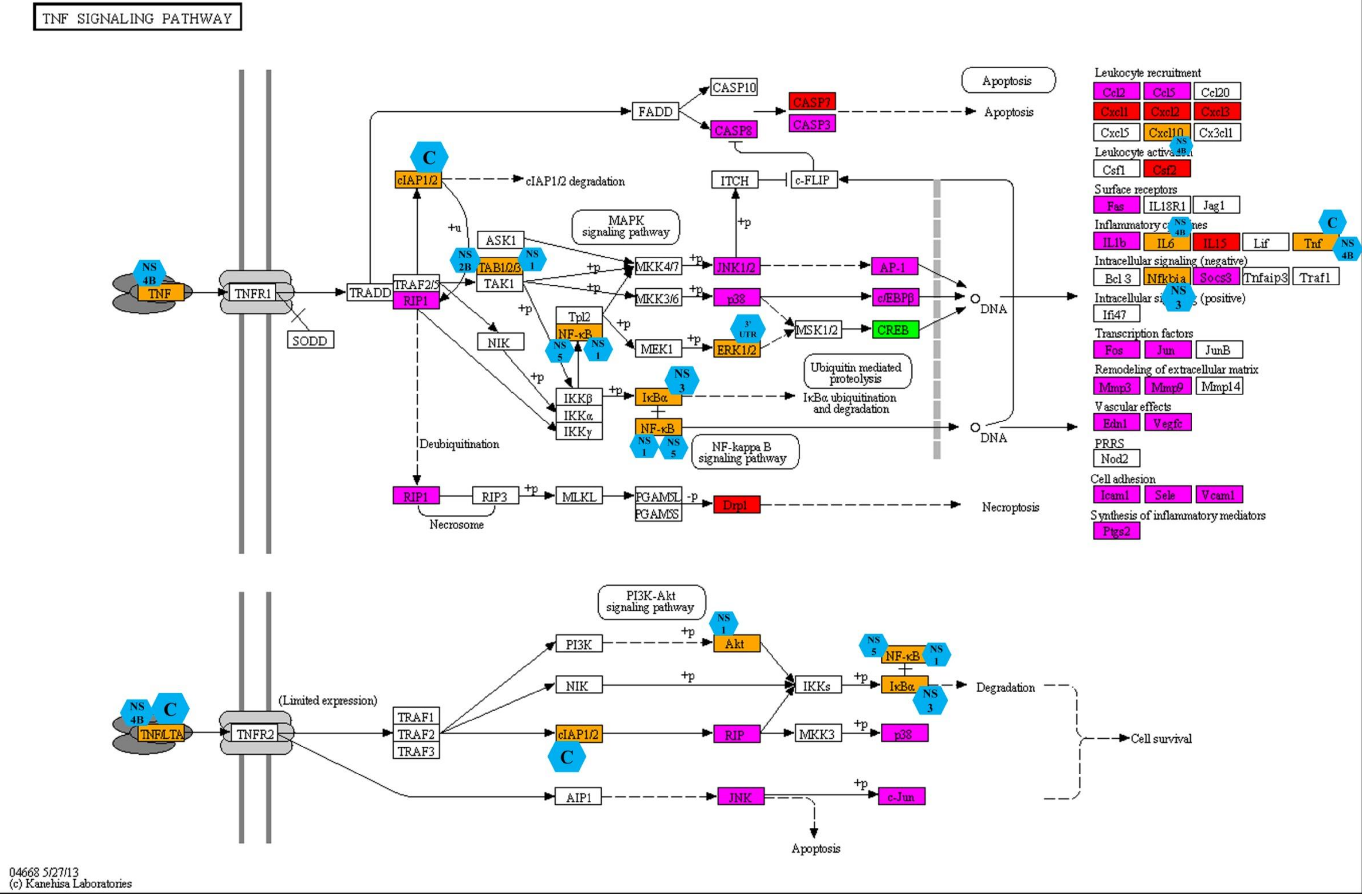
TNF SIGNALING PATHWAY
TNF
TNFR1
TRADD
TRAF2/5
RIP1
SODD
FADD
CASP10
CASP8
CASP7
CASP3
Apoptosis
cIAP1/2
cIAP1/2 degradation
ASK1
TAB1/2/3
TAK1
NIK
Tpl2
NF-κB
MAPK signaling pathway
MKK4/7
MKK3/6
MEK1
JNK1/2
p38
ERK1/2
ITCH
c-FLIP
AP-1
c/EBPβ
MSK1/2
CREB
DNA
IKKβ
IKKα
IKKγ
IκBα
Ubiquitin mediated proteolysis
IκBα ubiquitination and degradation
NF-kappa B signaling pathway
Deubiquitination
RIP3
MLKL
PGAM5L
PGAM5S
Drp1
Necrosome
Necroptosis
+u
+p
-p
Leukocyte recruitment
Ccl2
Ccl5
Ccl20
Cxcl1
Cxcl2
Cxcl3
Cxcl5
Cxcl10
Cx3cl1
Csf1
Csf2
Surface receptors
Fas
IL18R1
Jag1
IL1b
IL6
IL15
Lif
Tnf
Intracellular signaling (negative)
Bcl 3
Nfkbia
Socs3
Tnfaip3
Traf1
Ifi47
Transcription factors
Fos
Jun
JunB
Remodeling of extracellular matrix
Mmp3
Mmp9
Mmp14
Vascular effects
Edn1
Vegfc
PRRS
Nod2
Cell adhesion
Icam1
Sele
Vcam1
Synthesis of inflammatory mediators
Ptgs2
C
NS 4B
NS 2B
NS 1
NS 5
NS 3
3' UTR
TNF/LTA
TNFR2
(Limited expression)
TRAF1
TRAF2
TRAF3
PI3K
PI3K-Akt signaling pathway
Akt
IKKs
MKK3
RIP
AIP1
JNK
c-Jun
Degradation
Cell survival
04668 5/27/13
(c) Kanehisa Laboratories

C

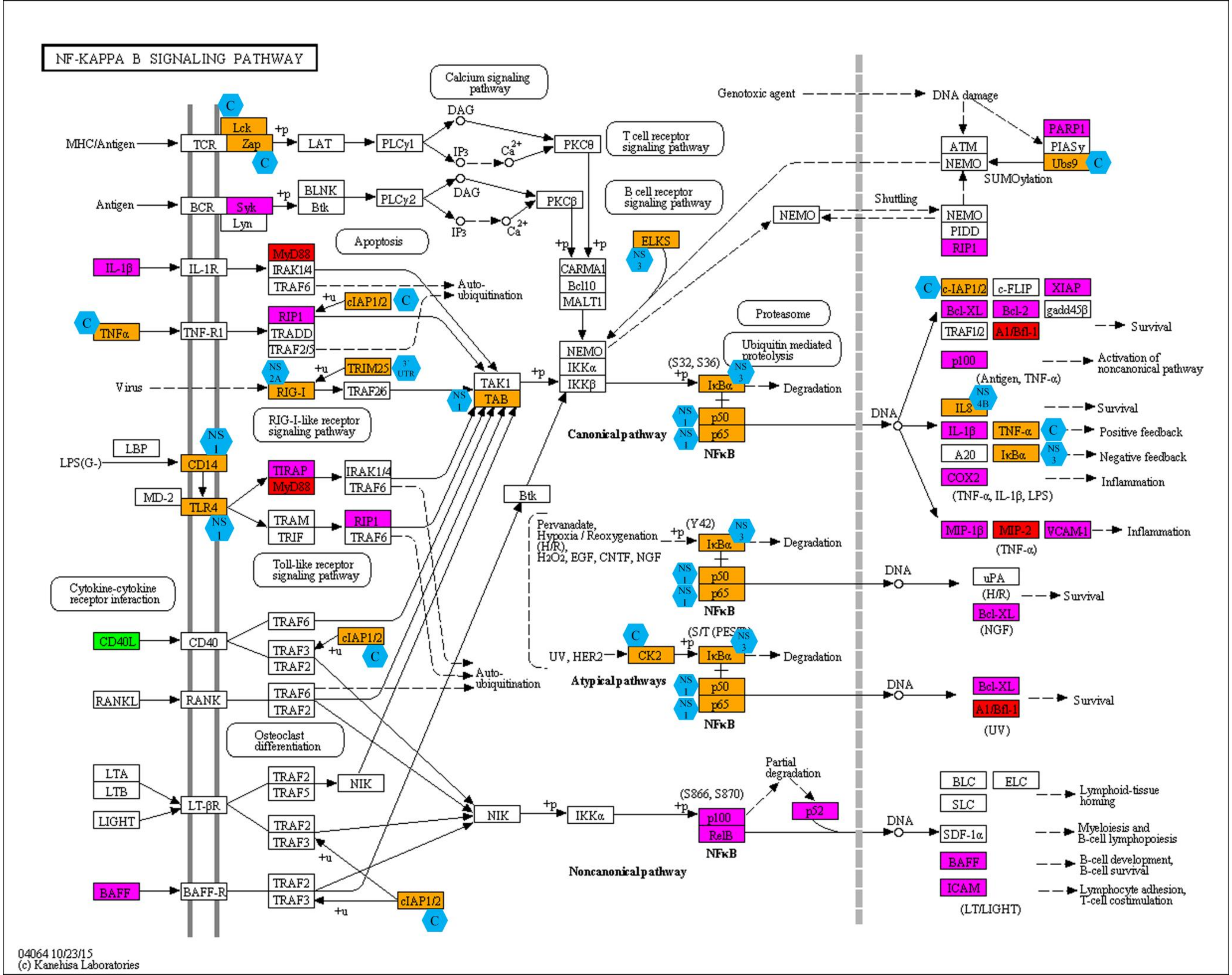
NF-KAPPA B SIGNALING PATHWAY
MHC/Antigen
TCR
Lck
Zap
LAT
PLCγ1
DAG
IP3
Ca2+
PKCθ
Calcium signaling pathway
T cell receptor signaling pathway
Antigen
BCR
Syk
Lyn
BLNK
Btk
PLCγ2
PKCβ
B cell receptor signaling pathway
Apoptosis
IL-1β
IL-1R
MyD88
IRAK1/4
TRAF6
Auto-ubiquitination
cIAP1/2
TNFα
TNF-R1
RIP1
TRADD
TRAF2/5
Virus
RIG-I
TRAF2/6
TRIM25
3' UTR
TAK1
TAB
RIG-I-like receptor signaling pathway
LBP
LPS(G-)
CD14
MD-2
TLR4
TIRAP
TRAM
TRIF
Toll-like receptor signaling pathway
Btk
CARMA1
Bcl10
MALT1
ELKS
NEMO
IKKα
IKKβ
Cytokine-cytokine receptor interaction
CD40L
CD40
TRAF3
TRAF2
RANKL
RANK
Osteoclast differentiation
LTA
LTB
LIGHT
LT-βR
TRAF5
NIK
BAFF
BAFF-R
Proteasome
Ubiquitin mediated proteolysis
(S32, S36)
IκBα
Degradation
p50
p65
NFκB
Canonical pathway
Pervanadate, Hypoxia / Reoxygenation (H/R), H2O2, EGF, CNTF, NGF
(Y42)
UV, HER2
CK2
(S/T (PEST))
Atypical pathways
IKKα
(S866, S870)
p100
RelB
Partial degradation
p52
Noncanonical pathway
Genotoxic agent
DNA damage
ATM
PARP1
PIASy
Ubs9
SUMOylation
Shuttling
PIDD
DNA
c-IAP1/2
c-FLIP
XIAP
Bcl-XL
Bcl-2
gadd45β
TRAF1/2
A1/Bfl-1
Survival
p100
(Antigen, TNF-α)
Activation of noncanonical pathway
IL8
IL-1β
TNF-α
Positive feedback
A20
Negative feedback
COX2
Inflammation
(TNF-α, IL-1β, LPS)
MIP-1β
MIP-2
VCAM-1
(TNF-α)
uPA
(H/R)
(NGF)
(UV)
BLC
ELC
SLC
Lymphoid-tissue homing
SDF-1α
Myeloiesis and B-cell lymphopoiesis
B-cell development, B-cell survival
ICAM
Lymphocyte adhesion, T-cell costimulation
(LT/LIGHT)
C
NS 1
NS 2A
NS 3
NS 4B
+p
+u
04064 10/23/15
(c) Kanehisa Laboratories

d

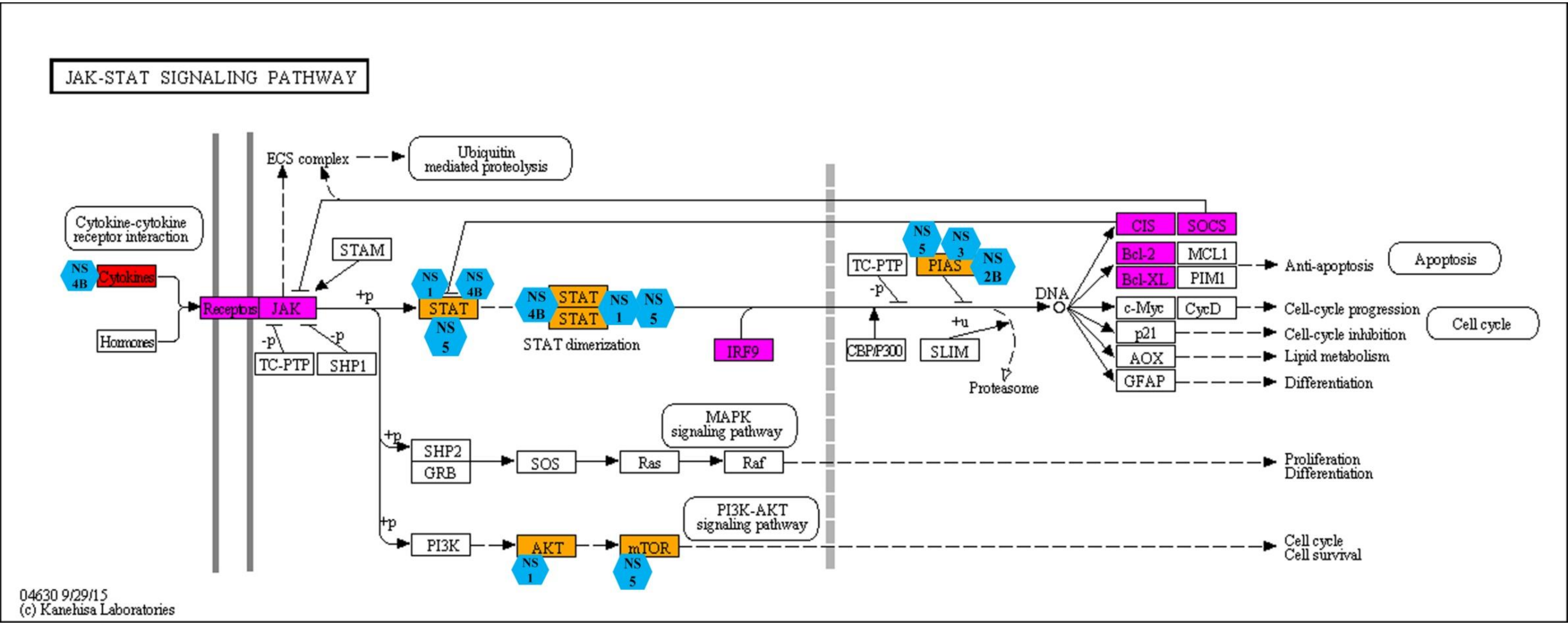
JAK-STAT SIGNALING PATHWAY
ECS complex
Ubiquitin mediated proteolysis
Cytokine-cytokine receptor interaction
NS 4B
Cytokines
Hormones
Receptors
JAK
STAM
-p
TC-PTP
SHP1
+p
NS 1
NS 4B
STAT
NS 5
STAT
STAT
STAT dimerization
NS 1
NS 5
IRF9
TC-PTP
CBP/P300
NS 5
NS 3
PIAS
NS 2B
+u
SLIM
Proteasome
DNA
CIS
SOCS
Bcl-2
MCL1
Bcl-XL
PIM1
c-Myc
CycD
p21
AOX
GFAP
Anti-apoptosis
Apoptosis
Cell-cycle progression
Cell cycle
Cell-cycle inhibition
Lipid metabolism
Differentiation
MAPK signaling pathway
SHP2
GRB
SOS
Ras
Raf
Proliferation
Differentiation
PI3K-AKT signaling pathway
PI3K
AKT
mTOR
Cell cycle
Cell survival
04630 9/29/15
(c) Kanehisa Laboratories

e

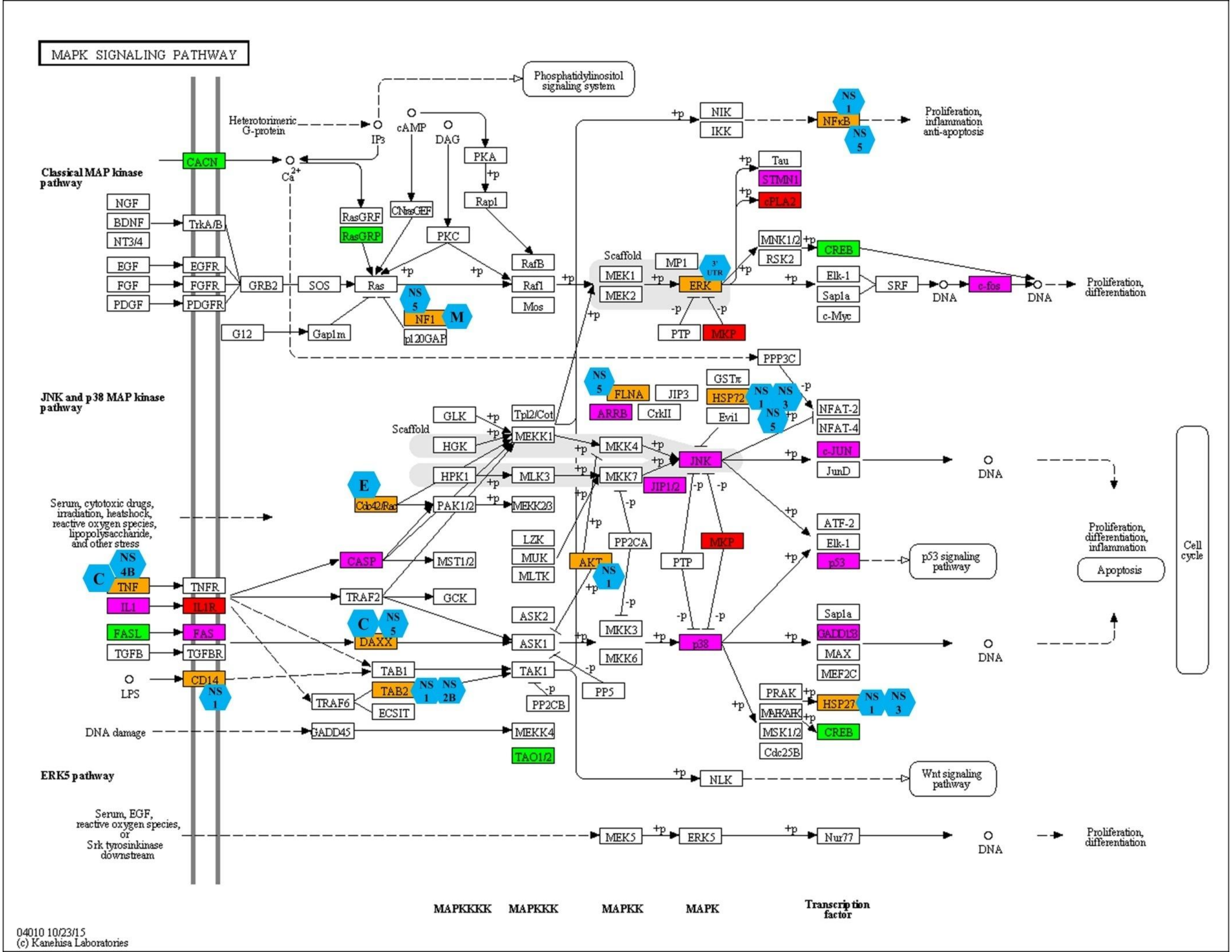

MAPK SIGNALING PATHWAY
Classical MAP kinase pathway
JNK and p38 MAP kinase pathway
ERK5 pathway
Phosphatidylinositol signaling system
Heterotorimeric G-protein
IP3
cAMP
DAG
CACN
PKA
Rap1
RasGRF
RasGRP
CNrasGEF
PKC
NGF
BDNF
NT3/4
TrkA/B
EGF
EGFR
FGF
FGFR
PDGF
PDGFR
GRB2
SOS
Ras
NF1
p120GAP
G12
Gap1m
RafB
Raf1
Mos
NIK
IKK
NFκB
Proliferation, inflammation anti-apoptosis
Tau
STMN1
cPLA2
MNK1/2
RSK2
CREB
Scaffold
MP1
MEK1
MEK2
ERK
PTP
MKP
Elk-1
Sap1a
c-Myc
SRF
c-fos
DNA
Proliferation, differentiation
PPP3C
GSTπ
HSP72
FLNA
JIP3
ARRB
CrkII
Evi1
NFAT-2
NFAT-4
GLK
Tpl2/Cot
MEKK1
HGK
HPK1
MLK3
MKK4
MKK7
JNK
JIP1/2
c-JUN
JunD
Cdc42/Rac
PAK1/2
MEKK2/3
ATF-2
Elk-1
p53
p53 signaling pathway
Serum, cytotoxic drugs, irradiation, heatshock, reactive oxygen species, lipopolysaccharide, and other stress
TNF
TNFR
IL1
IL1R
FASL
FAS
TGFB
TGFBR
LPS
CD14
CASP
MST1/2
TRAF2
GCK
LZK
MUK
MLTK
AKT
PP2CA
ASK2
DAXX
ASK1
MKK3
MKK6
p38
Sap1a
GADD153
MAX
MEF2C
TAB1
TAB2
TAK1
TRAF6
ECSIT
PP2CB
PP5
PRAK
MAPKAPK
MSK1/2
Cdc25B
HSP27
DNA damage
GADD45
MEKK4
TAO1/2
NLK
Wnt signaling pathway
Proliferation, differentiation, inflammation
Apoptosis
Cell cycle
Serum, EGF, reactive oxygen species, or Srk tyrosinkinase downstream
MEK5
ERK5
Nur77
MAPKKKK
MAPKKK
MAPKK
MAPK
Transcription factor
04010 10/23/15
(c) Kanehisa Laboratories

f

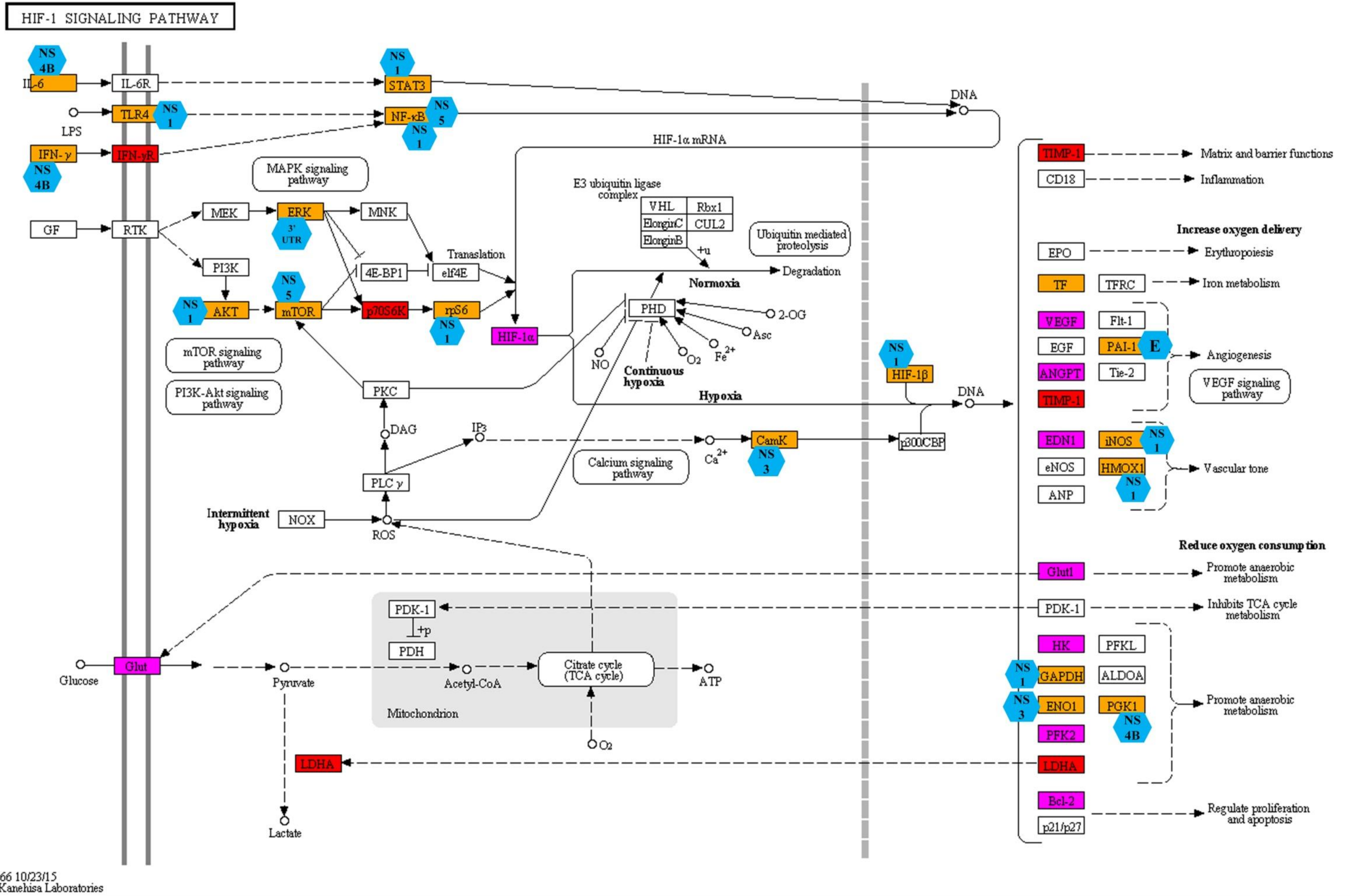
HIF-1 SIGNALING PATHWAY
IL-6
IL-6R
STAT3
DNA
LPS
TLR4
NF-κB
IFN-γ
IFN-γR
HIF-1α mRNA
MAPK signaling pathway
E3 ubiquitin ligase complex
VHL
Rbx1
ElonginC
CUL2
ElonginB
+u
Ubiquitin mediated proteolysis
GF
RTK
MEK
ERK
3' UTR
MNK
PI3K
4E-BP1
eIF4E
Translation
Normoxia
Degradation
AKT
mTOR
p70S6K
rpS6
PHD
2-OG
Asc
HIF-1α
NO
O2
Fe2+
Continuous hypoxia
mTOR signaling pathway
PI3K-Akt signaling pathway
PKC
HIF-1β
DNA
Hypoxia
DAG
IP3
Ca2+
CamK
p300/CBP
Calcium signaling pathway
PLCγ
Intermittent hypoxia
NOX
ROS
PDK-1
+p
PDH
Glucose
Glut
Pyruvate
Acetyl-CoA
Citrate cycle (TCA cycle)
ATP
Mitochondrion
O2
LDHA
Lactate
TIMP-1
Matrix and barrier functions
CD18
Inflammation
Increase oxygen delivery
EPO
Erythropoiesis
TF
TFRC
Iron metabolism
VEGF
Flt-1
EGF
PAI-1
Angiogenesis
ANGPT
Tie-2
VEGF signaling pathway
TIMP-1
EDN1
iNOS
eNOS
HMOX1
Vascular tone
ANP
Reduce oxygen consumption
Glut1
Promote anaerobic metabolism
PDK-1
Inhibits TCA cycle metabolism
HK
PFKL
GAPDH
ALDOA
ENO1
PGK1
Promote anaerobic metabolism
PFK2
LDHA
Bcl-2
p21/p27
Regulate proliferation and apoptosis
NS 4B
NS 1
NS 5
NS 3
E
04066 10/23/15
(c) Kanehisa Laboratories

g

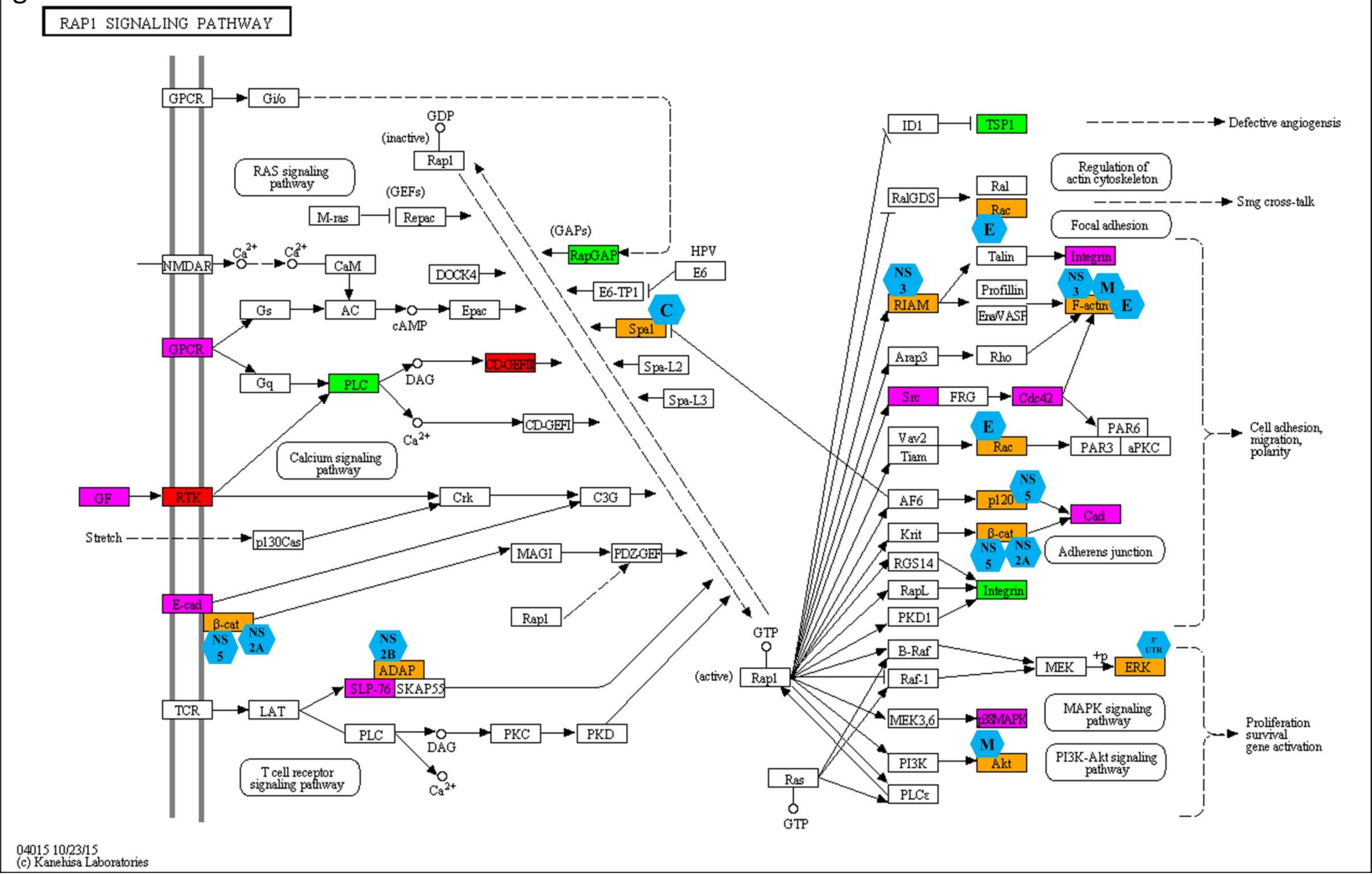
RAP1 SIGNALING PATHWAY
GPCR
Gi/o
GDP
(inactive)
Rap1
RAS signaling pathway
(GEFs)
M-ras
Repac
(GAPs)
RapGAP
HPV
E6
NMDAR
Ca2+
Ca2+
CaM
DOCK4
E6-TP1
Gs
AC
cAMP
Epac
C
Spa1
GPCR
Gq
PLC
DAG
CD-GEFII
Spa-L2
Spa-L3
Ca2+
CD-GEFI
Calcium signaling pathway
GF
RTK
Crk
C3G
Stretch
p130Cas
MAGI
PDZ-GEF
Rap1
E-cad
β-cat
NS 5
NS 2A
NS 2B
ADAP
SLP-76
SKAP55
TCR
LAT
PLC
DAG
PKC
PKD
Ca2+
T cell receptor signaling pathway
GTP
(active)
Rap1
Ras
GTP
ID1
TSP1
Defective angiogensis
Regulation of actin cytoskeleton
RalGDS
Ral
Rac
E
Smg cross-talk
Focal adhesion
Talin
Integrin
NS 3
RIAM
Profillin
Ena/VASP
NS 3
M
F-actin
E
Arap3
Rho
Src
FRG
Cdc42
PAR6
Vav2
Tiam
E
Rac
PAR3
aPKC
Cell adhesion, migration, polarity
AF6
p120
NS 5
Cad
Krit
β-cat
NS 5
NS 2A
Adherens junction
RGS14
RapL
Integrin
PKD1
B-Raf
Raf-1
MEK
+p
ERK
3' UTR
MAPK signaling pathway
MEK3,6
p38MAPK
PI3K
M
Akt
PI3K-Akt signaling pathway
PLCε
Proliferation survival gene activation
04015 10/23/15
(c) Kanehisa Laboratories

h

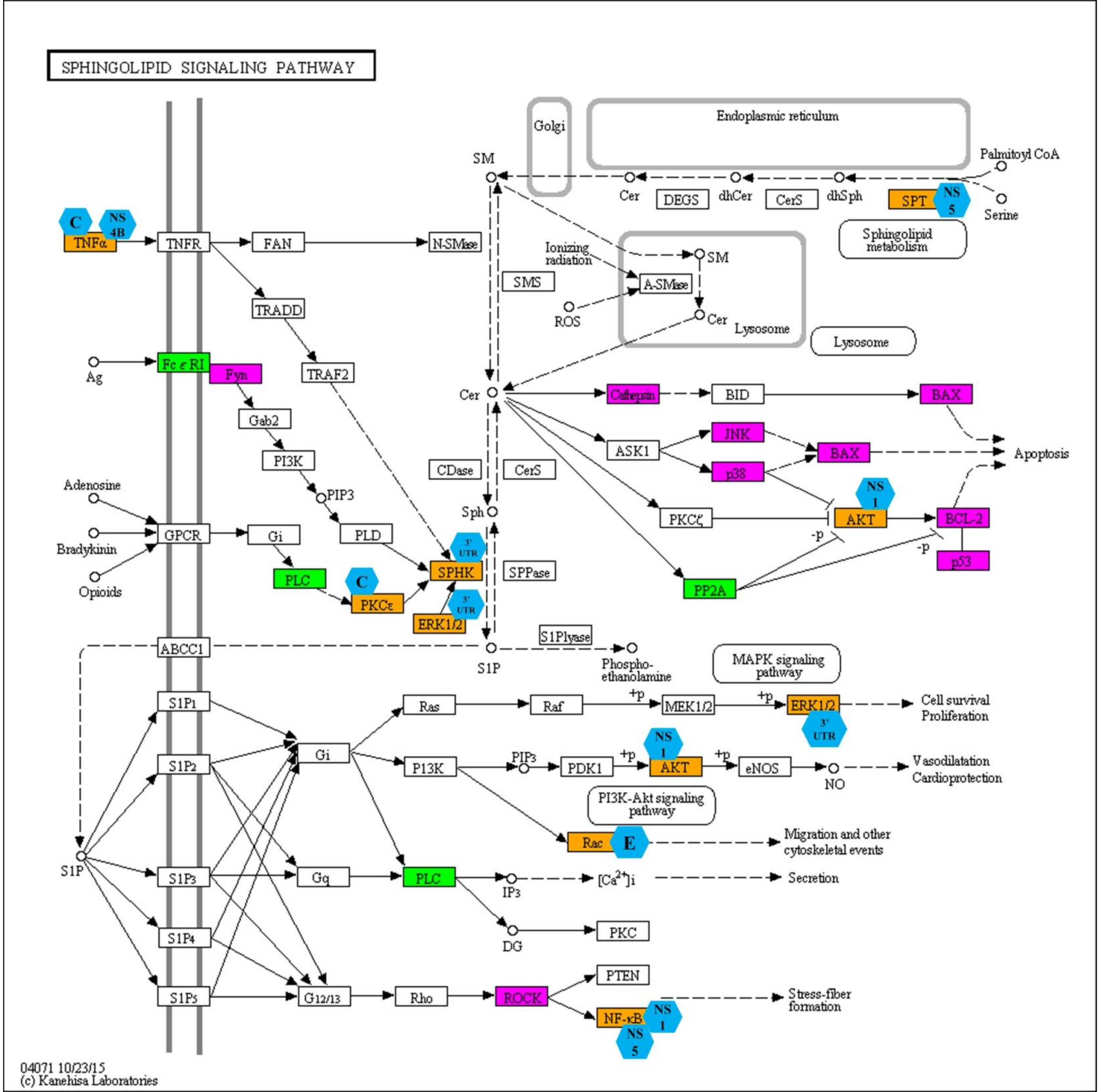
SPHINGOLIPID SIGNALING PATHWAY
Golgi
Endoplasmic reticulum
SM
Cer
DEGS
dhCer
CerS
dhSph
Palmitoyl CoA
SPT
NS 5
Serine
Sphingolipid metabolism
C
NS 4B
TNFα
TNFR
FAN
N-SMase
Ionizing radiation
SM
A-SMase
SMS
ROS
Cer
Lysosome
Lysosome
TRADD
Ag
Fc ε RI
Fyn
TRAF2
Cer
Cathepsin
BID
BAX
Gab2
ASK1
JNK
BAX
Apoptosis
PI3K
CDase
CerS
p38
Adenosine
PIP3
NS 1
Sph
PKCζ
AKT
BCL-2
GPCR
Gi
PLD
-p
-p
Bradykinin
3' UTR
SPHK
SPPase
p53
Opioids
PLC
C
PKCε
3' UTR
ERK1/2
PP2A
ABCC1
S1Plyase
S1P
Phospho-ethanolamine
MAPK signaling pathway
S1P1
Ras
Raf
+p
MEK1/2
+p
ERK1/2
Cell survival
Proliferation
3' UTR
Gi
NS 1
S1P2
P13K
PIP3
PDK1
+p
AKT
+p
eNOS
NO
Vasodilatation
Cardioprotection
PI3K-Akt signaling pathway
Rac
E
Migration and other cytoskeletal events
S1P
S1P3
Gq
PLC
IP3
[Ca2+]i
Secretion
S1P4
DG
PKC
PTEN
S1P5
G12/13
Rho
ROCK
NF-κB
NS 1
NS 5
Stress-fiber formation
04071 10/23/15
(c) Kanehisa Laboratories

i

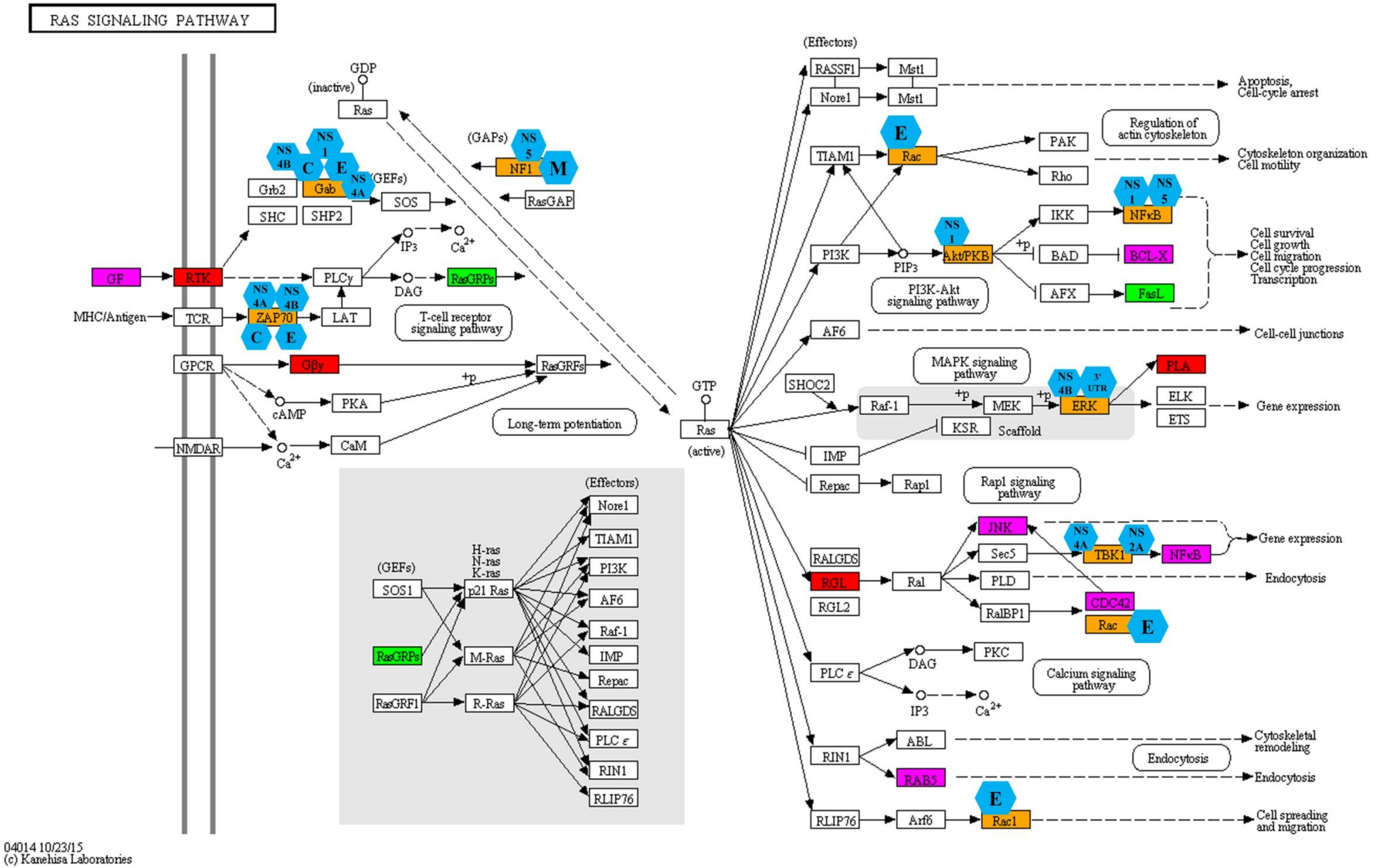

RAS SIGNALING PATHWAY
GDP
(inactive)
Ras
(GAPs)
NF1
RasGAP
Grb2
Gab
(GEFs)
SOS
SHC
SHP2
GF
RTK
PLCγ
IP3
Ca2+
DAG
RasGRPs
MHC/Antigen
TCR
ZAP70
LAT
T-cell receptor signaling pathway
GPCR
Gβγ
RasGRFs
+p
cAMP
PKA
NMDAR
CaM
Long-term potentiation
(Effectors)
Nore1
TIAM1
PI3K
AF6
Raf-1
IMP
Repac
RALGDS
PLC ε
RIN1
RLIP76
(GEFs)
SOS1
RasGRPs
RasGRF1
H-ras
N-ras
K-ras
p21 Ras
M-Ras
R-Ras
GTP
Ras
(active)
RASSF1
Mst1
Apoptosis, Cell-cycle arrest
Rac
PAK
Rho
Regulation of actin cytoskeleton
Cytoskeleton organization
Cell motility
IKK
NFκB
PIP3
Akt/PKB
BAD
BCL-X
AFX
FasL
PI3K-Akt signaling pathway
Cell survival
Cell growth
Cell migration
Cell cycle progression
Transcription
Cell-cell junctions
MAPK signaling pathway
SHOC2
MEK
ERK
3' UTR
PLA
ELK
ETS
Gene expression
KSR
Scaffold
Rap1
Rap1 signaling pathway
JNK
Sec5
TBK1
NFκB
Gene expression
RGL
RGL2
Ral
PLD
Endocytosis
RalBP1
CDC42
Rac
PKC
Calcium signaling pathway
ABL
RAB5
Cytoskeletal remodeling
Endocytosis
Arf6
Rac1
Cell spreading and migration
NS 1
NS 4A
NS 4B
NS 5
NS 2A
C
E
M
04014 10/23/15
(c) Kanehisa Laboratories

j

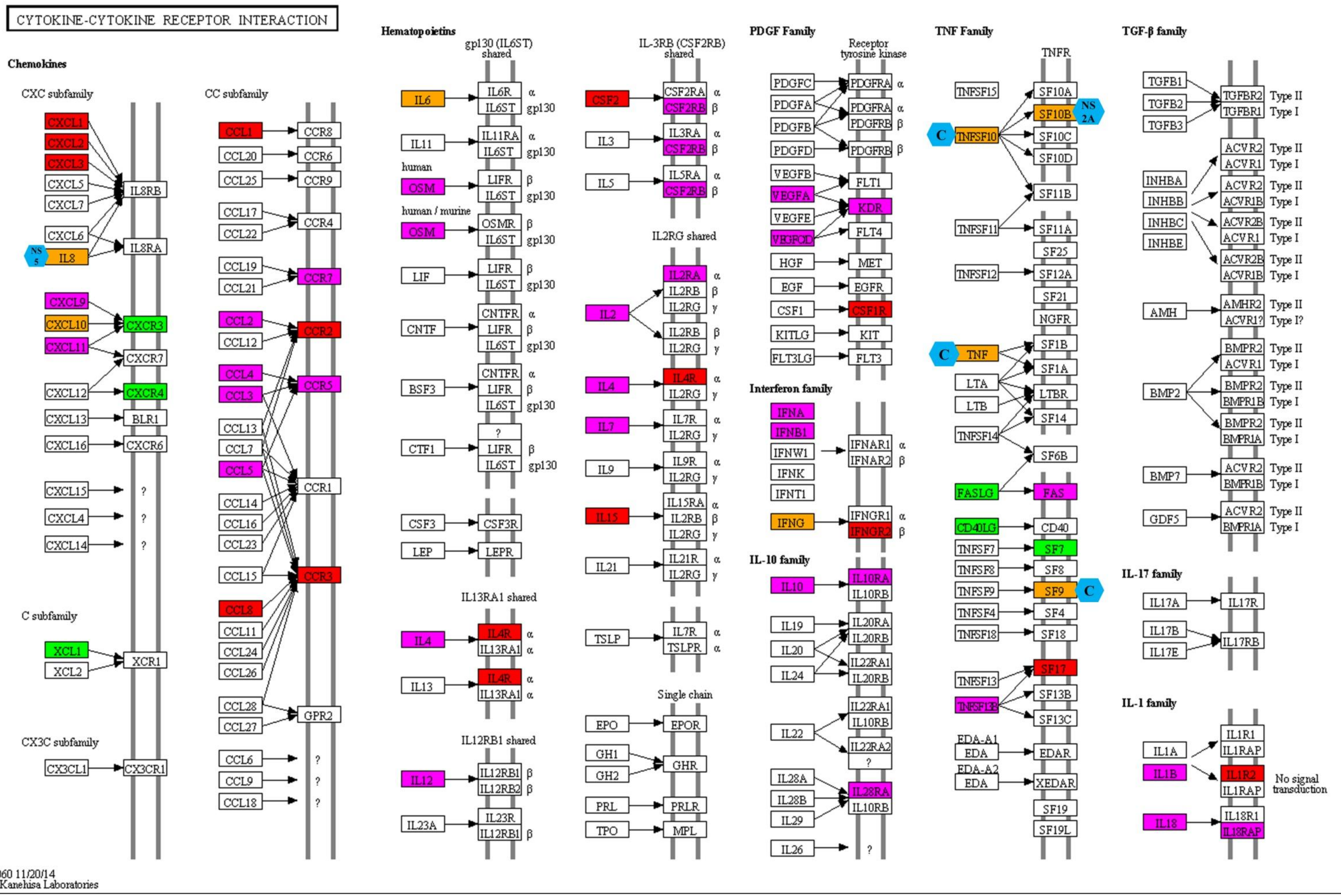

CYTOKINE-CYTOKINE RECEPTOR INTERACTION
Chemokines
CXC subfamily
CXCL1
CXCL2
CXCL3
CXCL5
CXCL7
CXCL6
IL8
NS 5
IL8RB
IL8RA
CXCL9
CXCL10
CXCL11
CXCR3
CXCR7
CXCL12
CXCR4
CXCL13
BLR1
CXCL16
CXCR6
CXCL15
CXCL4
CXCL14
?
C subfamily
XCL1
XCL2
XCR1
CX3C subfamily
CX3CL1
CX3CR1
CC subfamily
CCL1
CCR8
CCL20
CCR6
CCL25
CCR9
CCL17
CCL22
CCR4
CCL19
CCL21
CCR7
CCL2
CCL12
CCR2
CCL4
CCL3
CCR5
CCL13
CCL7
CCL5
CCR1
CCL14
CCL16
CCL23
CCL15
CCR3
CCL8
CCL11
CCL24
CCL26
CCL28
CCL27
GPR2
CCL6
CCL9
CCL18
Hematopoietins
gp130 (IL6ST) shared
IL6
IL6R
IL6ST
IL11
IL11RA
human
OSM
LIFR
human / murine
OSMR
LIF
CNTF
CNTFR
BSF3
CTF1
CSF3
CSF3R
LEP
LEPR
IL13RA1 shared
IL4
IL4R
IL13RA1
IL13
IL12RB1 shared
IL12
IL12RB1
IL12RB2
IL23A
IL23R
IL-3RB (CSF2RB) shared
CSF2
CSF2RA
CSF2RB
IL3
IL3RA
IL5
IL5RA
IL2RG shared
IL2
IL2RA
IL2RB
IL2RG
IL7
IL7R
IL9
IL9R
IL15
IL15RA
IL21
IL21R
TSLP
TSLPR
Single chain
EPO
EPOR
GH1
GH2
GHR
PRL
PRLR
TPO
MPL
PDGF Family
Receptor tyrosine kinase
PDGFC
PDGFA
PDGFB
PDGFD
PDGFRA
PDGFRB
VEGFB
VEGFA
VEGFE
VEGFC/D
FLT1
KDR
FLT4
HGF
MET
EGF
EGFR
CSF1
CSF1R
KITLG
KIT
FLT3LG
FLT3
Interferon family
IFNA
IFNB1
IFNW1
IFNK
IFNT1
IFNAR1
IFNAR2
IFNG
IFNGR1
IFNGR2
IL-10 family
IL10
IL10RA
IL10RB
IL19
IL20
IL24
IL20RA
IL20RB
IL22RA1
IL22
IL22RA2
IL28A
IL28B
IL29
IL28RA
IL26
TNF Family
TNFR
TNFSF15
SF10A
SF10B
NS 2A
C
TNFSF10
SF10C
SF10D
SF11B
TNFSF11
SF11A
SF25
TNFSF12
SF12A
SF21
NGFR
TNF
SF1B
SF1A
LTA
LTB
LTBR
TNFSF14
SF14
SF6B
FASLG
FAS
CD40LG
CD40
TNFSF7
SF7
TNFSF8
SF8
TNFSF9
SF9
TNFSF4
SF4
TNFSF18
SF18
TNFSF13
SF17
TNFSF13B
SF13B
SF13C
EDA-A1
EDA
EDAR
EDA-A2
XEDAR
SF19
SF19L
TGF-β family
TGFB1
TGFB2
TGFB3
TGFBR2
TGFBR1
Type II
Type I
INHBA
INHBB
INHBC
INHBE
ACVR2
ACVR1
ACVR1B
ACVR2B
AMH
AMHR2
ACVR1?
Type I?
BMP2
BMPR2
BMPR1B
BMPR1A
BMP7
GDF5
IL-17 family
IL17A
IL17R
IL17B
IL17E
IL17RB
IL-1 family
IL1A
IL1R1
IL1RAP
IL1B
IL1R2
No signal transduction
IL18
IL18R1
IL18RAP
α
β
γ
04060 11/20/14
(c) Kanehisa Laboratories

k

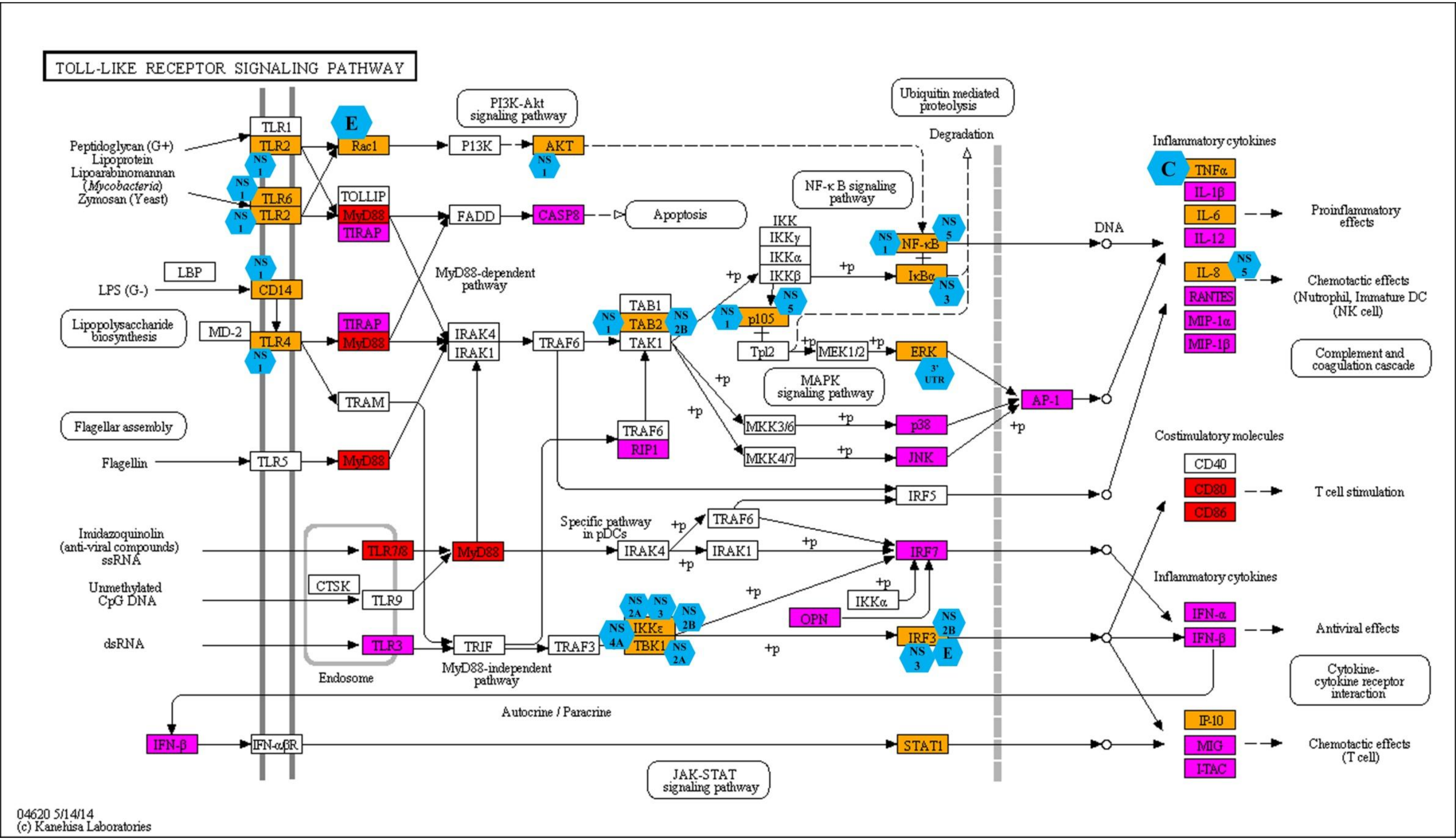

TOLL-LIKE RECEPTOR SIGNALING PATHWAY
Peptidoglycan (G+)
Lipoprotein
Lipoarabinomannan
(Mycobacteria)
Zymosan (Yeast)
LPS (G-)
LBP
Lipopolysaccharide biosynthesis
MD-2
Flagellar assembly
Flagellin
Imidazoquinolin (anti-viral compounds) ssRNA
Unmethylated CpG DNA
dsRNA
TLR1
TLR2
TLR6
CD14
TLR4
TLR5
TLR7/8
TLR9
TLR3
CTSK
Endosome
Rac1
TOLLIP
MyD88
TIRAP
TRAM
TRIF
IFN-β
IFN-α/βR
PI3K-Akt signaling pathway
P13K
AKT
FADD
CASP8
Apoptosis
MyD88-dependent pathway
MyD88-independent pathway
IRAK4
IRAK1
TRAF6
TAB1
TAB2
TAK1
RIP1
TRAF3
IKKε
TBK1
Specific pathway in pDCs
IKK
IKKγ
IKKα
IKKβ
NF-κB signaling pathway
NF-κB
IκBα
p105
Tpl2
MEK1/2
ERK
MAPK signaling pathway
MKK3/6
MKK4/7
p38
JNK
AP-1
IRF5
IRF7
IRF3
OPN
STAT1
Ubiquitin mediated proteolysis
Degradation
DNA
Autocrine / Paracrine
JAK-STAT signaling pathway
+p
NS 1
NS 2A
NS 2B
NS 3
NS 4A
NS 5
E
C
3' UTR
Inflammatory cytokines
TNFα
IL-1β
IL-6
IL-12
IL-8
RANTES
MIP-1α
MIP-1β
Proinflammatory effects
Chemotactic effects (Nutrophil, Immature DC (NK cell)
Complement and coagulation cascade
Costimulatory molecules
CD40
CD80
CD86
T cell stimulation
IFN-α
IFN-β
Antiviral effects
Cytokine-cytokine receptor interaction
IP-10
MIG
I-TAC
Chemotactic effects (T cell)
04620 5/14/14
(c) Kanehisa Laboratories

I

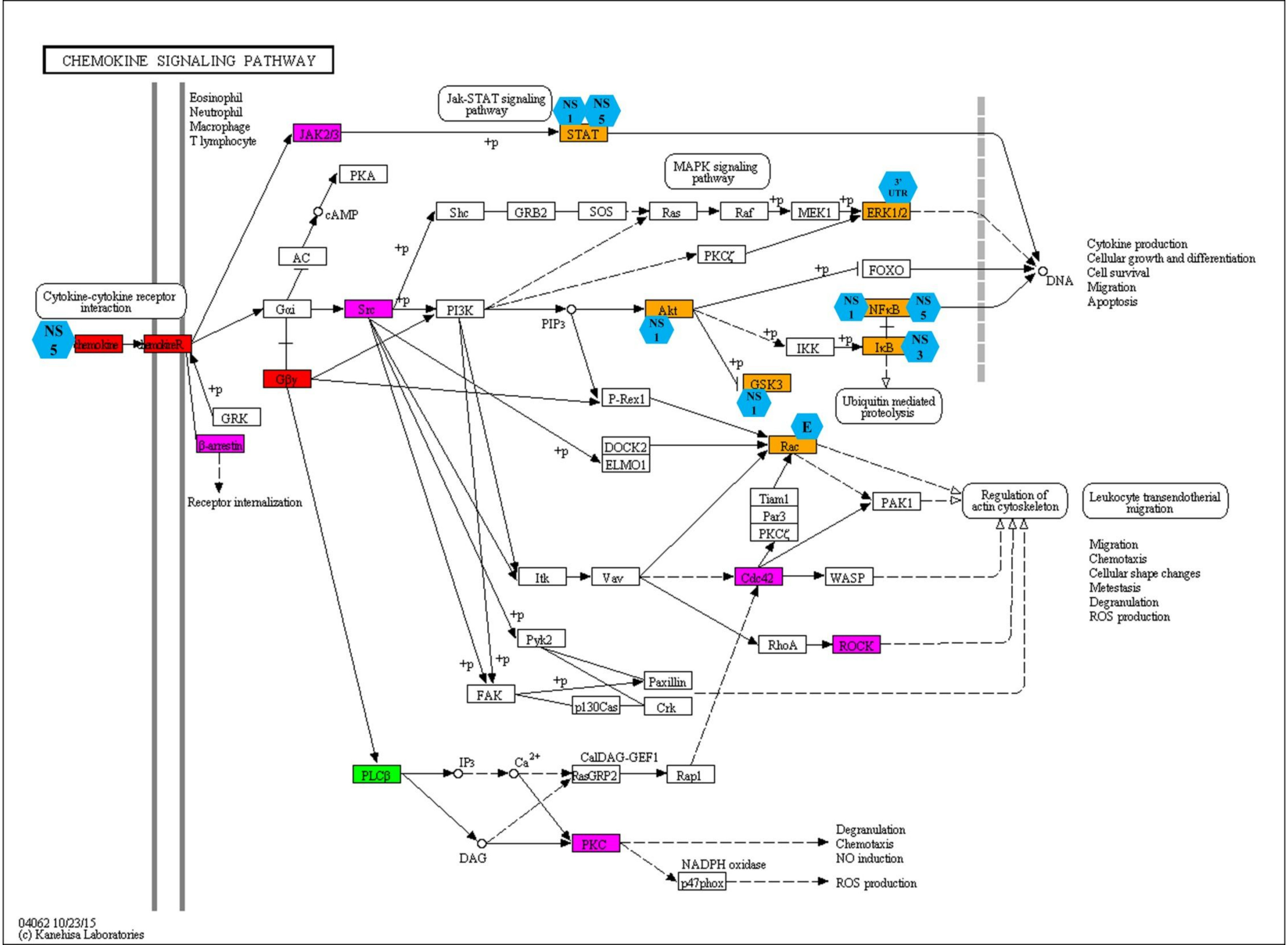
CHEMOKINE SIGNALING PATHWAY
Eosinophil
Neutrophil
Macrophage
T lymphocyte
Jak-STAT signaling pathway
NS 1
NS 5
JAK2/3
+p
STAT
MAPK signaling pathway
PKA
cAMP
AC
Shc
GRB2
SOS
Ras
Raf
MEK1
3' UTR
ERK1/2
PKCζ
FOXO
Cytokine-cytokine receptor interaction
chemokine
chemokineR
Gαi
Src
PI3K
PIP3
Akt
NFκB
IKK
IκB
NS 3
GSK3
Cytokine production
Cellular growth and differentiation
Cell survival
Migration
Apoptosis
DNA
Gβγ
GRK
β-arrestin
Receptor internalization
P-Rex1
Ubiquitin mediated proteolysis
E
Rac
DOCK2
ELMO1
Tiam1
Par3
PAK1
Regulation of actin cytoskeleton
Leukocyte transendothelial migration
Itk
Vav
Cdc42
WASP
RhoA
ROCK
Migration
Chemotaxis
Cellular shape changes
Metestasis
Degranulation
ROS production
Pyk2
Paxillin
FAK
p130Cas
Crk
PLCβ
IP3
Ca2+
CalDAG-GEF1
RasGRP2
Rap1
DAG
PKC
Degranulation
Chemotaxis
NO induction
NADPH oxidase
p47phox
ROS production
04062 10/23/15
(c) Kanehisa Laboratories

m

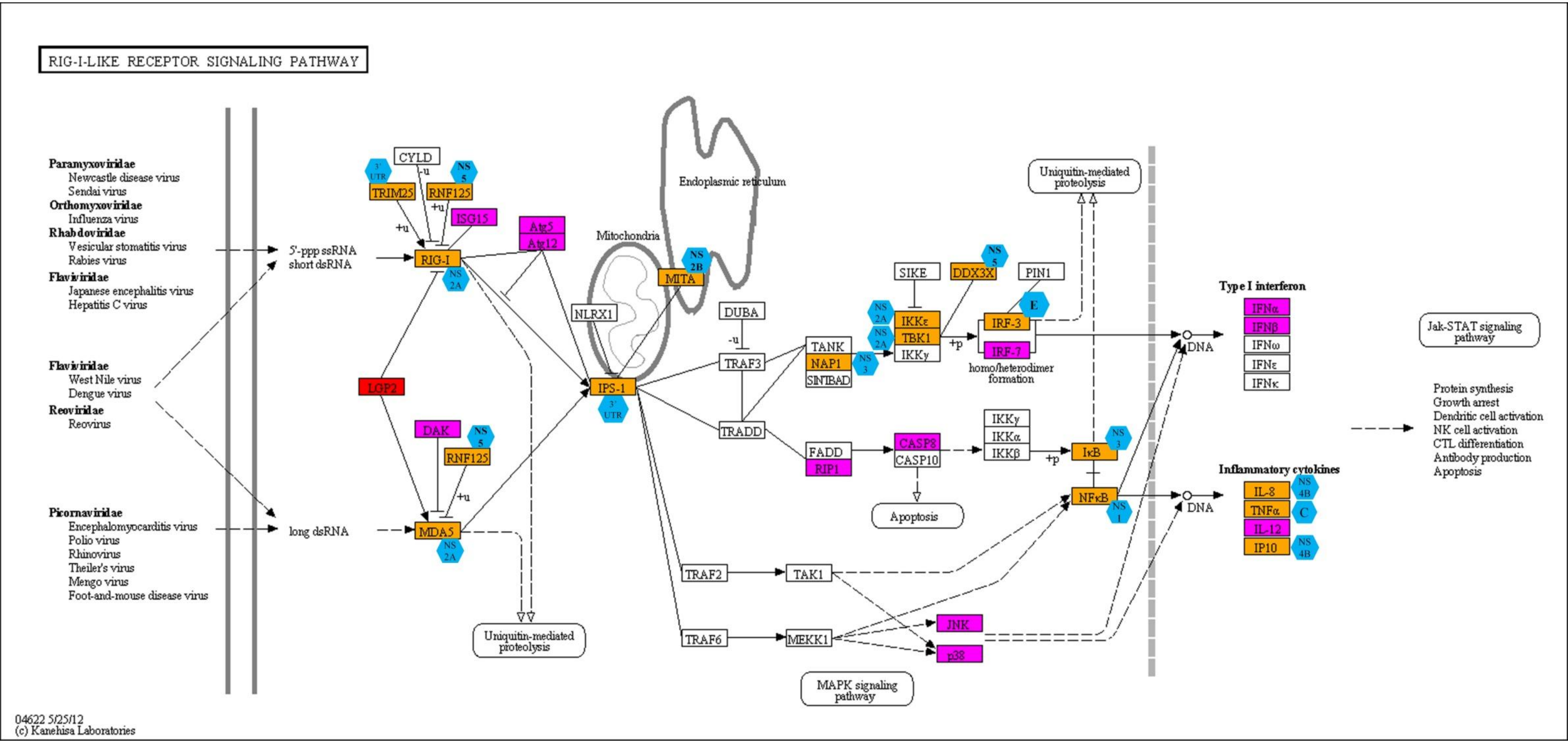

RIG-I-LIKE RECEPTOR SIGNALING PATHWAY
Paramyxoviridae
Newcastle disease virus
Sendai virus
Orthomyxoviridae
Influenza virus
Rhabdoviridae
Vesicular stomatitis virus
Rabies virus
Flaviviridae
Japanese encephalitis virus
Hepatitis C virus
Flaviviridae
West Nile virus
Dengue virus
Reoviridae
Reovirus
Picornaviridae
Encephalomyocarditis virus
Polio virus
Rhinovirus
Theiler's virus
Mengo virus
Foot-and-mouth disease virus
5'-ppp ssRNA
short dsRNA
long dsRNA
CYLD
TRIM25
RNF125
ISG15
RIG-I
Atg5
Atg12
LGP2
DAK
RNF125
MDA5
Mitochondria
Endoplasmic reticulum
MITA
NLRX1
IPS-1
Uniquitin-mediated proteolysis
DUBA
TRAF3
TRADD
TANK
NAP1
SINTBAD
FADD
RIP1
CASP8
CASP10
Apoptosis
SIKE
IKKε
TBK1
IKKγ
DDX3X
PIN1
IRF-3
IRF-7
homo/heterodimer formation
IKKγ
IKKα
IKKβ
IκB
NFκB
TRAF2
TAK1
TRAF6
MEKK1
JNK
p38
MAPK signaling pathway
Uniquitin-mediated proteolysis
DNA
DNA
Type I interferon
IFNα
IFNβ
IFNω
IFNε
IFNκ
Inflammatory cytokines
IL-8
TNFα
IL-12
IP10
Jak-STAT signaling pathway
Protein synthesis
Growth arrest
Dendritic cell activation
NK cell activation
CTL differentiation
Antibody production
Apoptosis
04622 5/25/12
(c) Kanehisa Laboratories

n

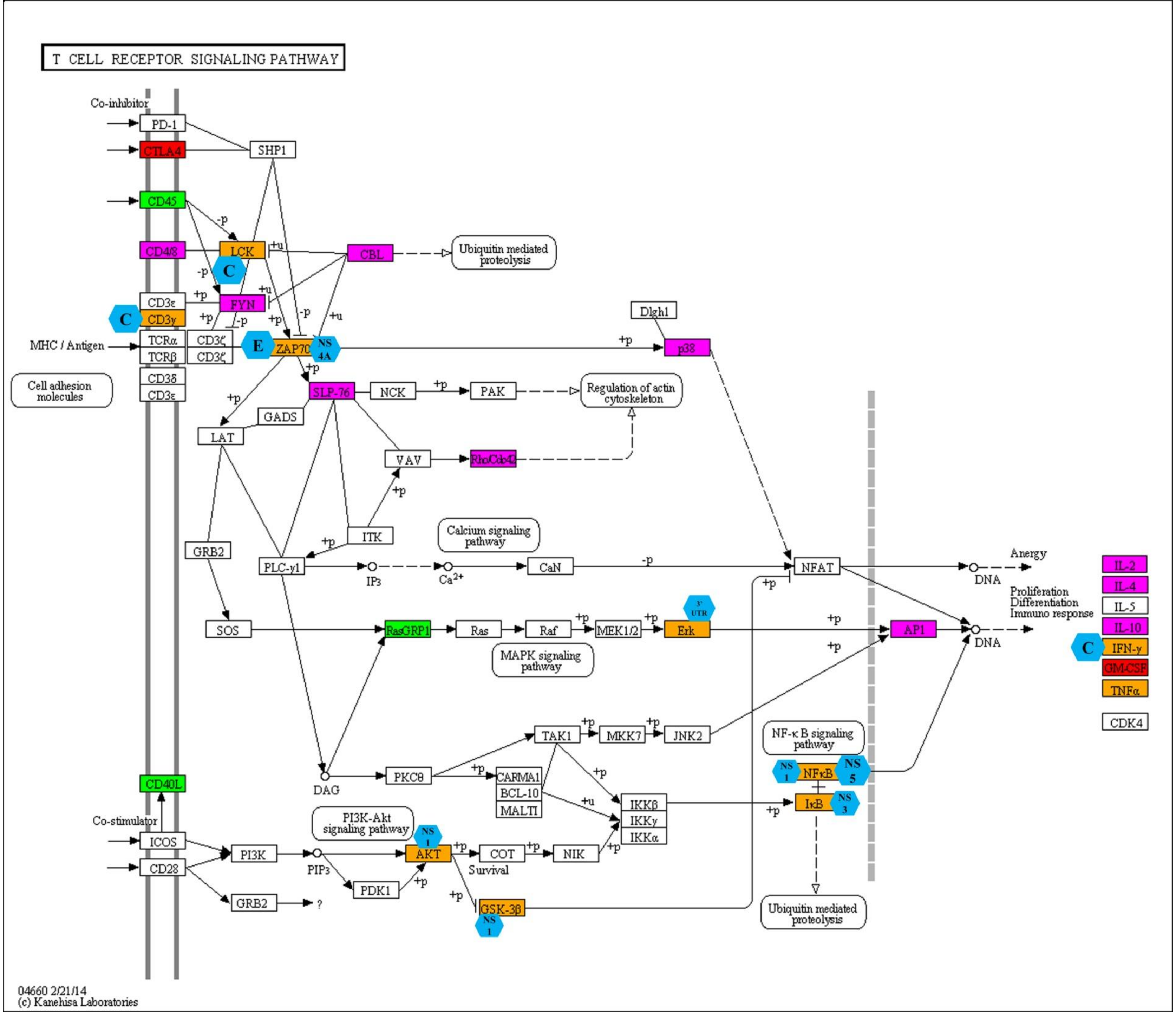

T CELL RECEPTOR SIGNALING PATHWAY
Co-inhibitor
PD-1
CTLA4
SHP1
CD45
CD4/8
LCK
CBL
Ubiquitin mediated proteolysis
CD3ε
CD3γ
FYN
TCRα
TCRβ
CD3ζ
ZAP70
NS 4A
MHC / Antigen
Dlgh1
p38
CD3δ
CD3ε
Cell adhesion molecules
SLP-76
NCK
PAK
Regulation of actin cytoskeleton
GADS
LAT
VAV
Rho/Cdc42
ITK
GRB2
PLC-γ1
IP3
Ca2+
CaN
Calcium signaling pathway
NFAT
DNA
Anergy
Proliferation
Differentiation
Immuno response
IL-2
IL-4
IL-5
IL-10
IFN-γ
GM-CSF
TNFα
CDK4
SOS
RasGRP1
Ras
Raf
MEK1/2
Erk
3' UTR
AP1
MAPK signaling pathway
TAK1
MKK7
JNK2
NF-κB signaling pathway
NFκB
NS 1
NS 5
IκB
NS 3
DAG
PKCθ
CARMA1
BCL-10
MALT1
IKKβ
IKKγ
IKKα
CD40L
Co-stimulator
ICOS
CD28
PI3K
PIP3
PI3K-Akt signaling pathway
AKT
COT
NIK
Survival
PDK1
GRB2
GSK-3β
Ubiquitin mediated proteolysis
C
E
04660 2/21/14
(c) Kanehisa Laboratories

O

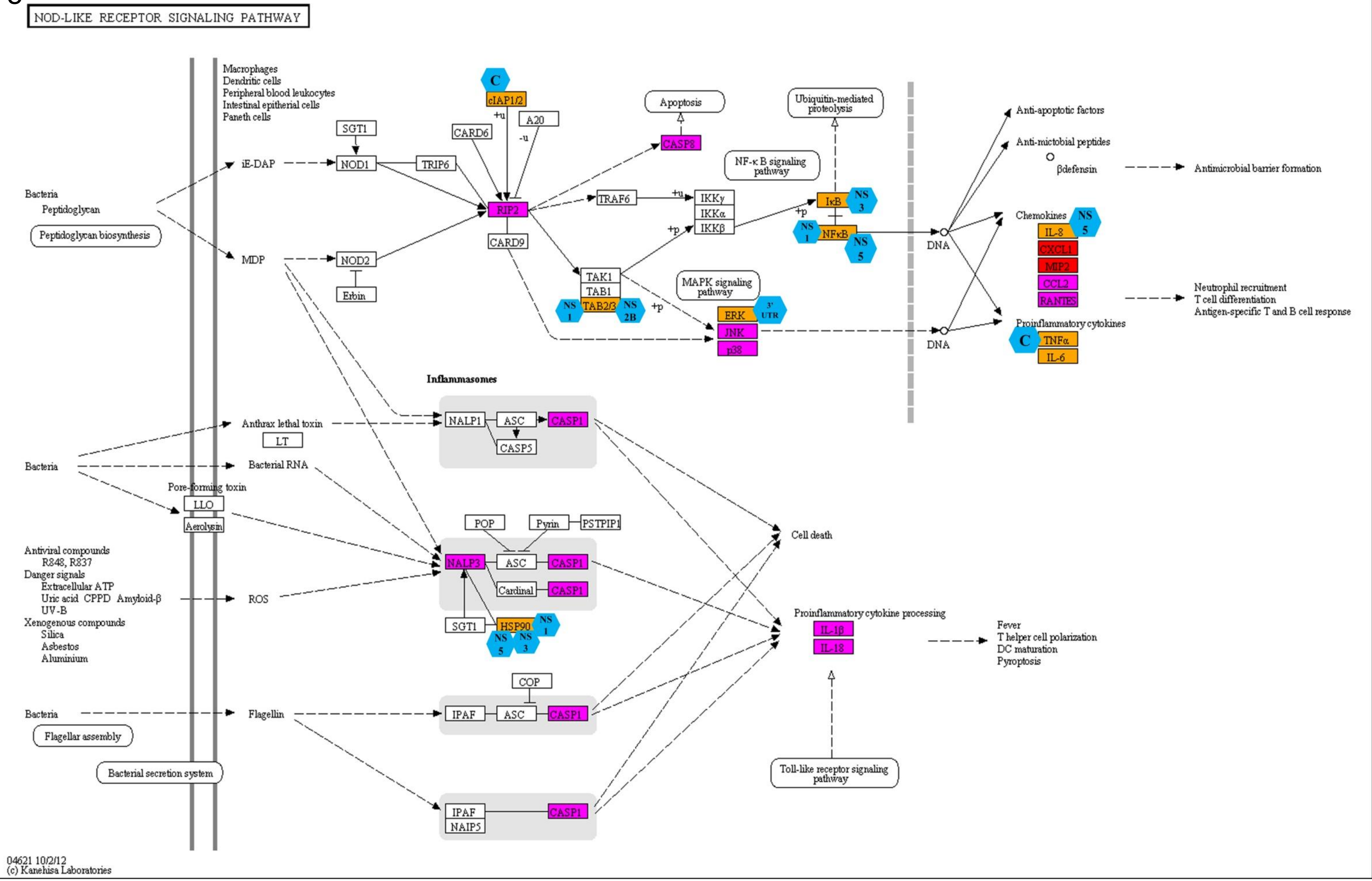
NOD-LIKE RECEPTOR SIGNALING PATHWAY
Macrophages
Dendritic cells
Peripheral blood leukocytes
Intestinal epithelial cells
Paneth cells
Bacteria
Peptidoglycan
Peptidoglycan biosynthesis
iE-DAP
MDP
SGT1
NOD1
TRIP6
NOD2
Erbin
CARD6
C
cIAP1/2
+u
A20
-u
RIP2
CARD9
Apoptosis
CASP8
TRAF6
+u
IKKγ
IKKα
IKKβ
+p
NF-κ B signaling pathway
Ubiquitin-mediated proteolysis
IκB
NS 3
NS 1
NFκB
NS 5
+p
TAK1
TAB1
TAB2/3
NS 1
NS 2B
+p
MAPK signaling pathway
ERK
3' UTR
JNK
p38
DNA
DNA
Anti-apoptotic factors
Anti-mictobial peptides
βdefensin
Antimicrobial barrier formation
Chemokines
NS 5
IL-8
CXCL1
MIP2
CCL2
RANTES
Neutrophil recruitment
T cell differentiation
Antigen-specific T and B cell response
Proinflammatory cytokines
C
TNFα
IL-6
Inflammasomes
Anthrax lethal toxin
LT
NALP1
ASC
CASP1
CASP5
Bacteria
Bacterial RNA
Pore-forming toxin
LLO
Aerolysin
POP
Pyrin
PSTPIP1
NALP3
ASC
CASP1
Cardinal
CASP1
Antiviral compounds
R848, R837
Danger signals
Extracellular ATP
Uric acid CPPD Amyloid-β
UV-B
Xenogenous compounds
Silica
Asbestos
Aluminium
ROS
SGT1
HSP90
NS 1
NS 5
NS 3
Cell death
Proinflammatory cytokine processing
IL-1β
IL-18
Fever
T helper cell polarization
DC maturation
Pyroptosis
Bacteria
Flagellar assembly
Bacterial secretion system
Flagellin
COP
IPAF
ASC
CASP1
IPAF
NAIP5
CASP1
Toll-like receptor signaling pathway
04621 10/2/12
(c) Kanehisa Laboratories

p

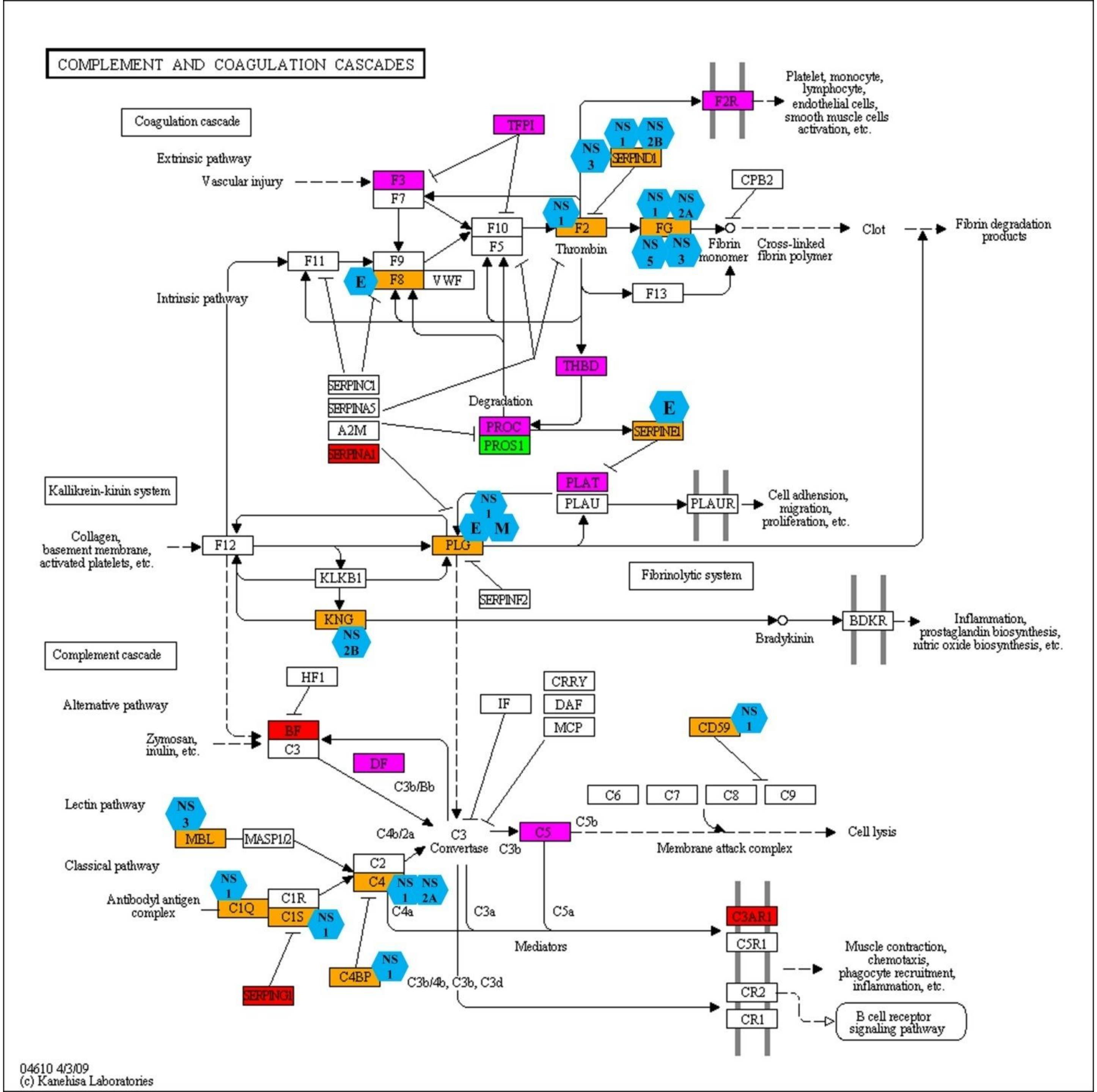
COMPLEMENT AND COAGULATION CASCADES
Coagulation cascade
Extrinsic pathway
Vascular injury
F3
F7
TFPI
F10
F5
F2
Thrombin
F2R
Platelet, monocyte, lymphocyte, endothelial cells, smooth muscle cells activation, etc.
SERPIND1
CPB2
FG
Fibrin monomer
Cross-linked fibrin polymer
Clot
Fibrin degradation products
F11
F9
F8
VWF
F13
Intrinsic pathway
THBD
SERPINC1
SERPINA5
A2M
SERPINA1
Degradation
PROC
PROS1
SERPINE1
PLAT
PLAU
PLAUR
Cell adhesion, migration, proliferation, etc.
Kallikrein-kinin system
Collagen, basement membrane, activated platelets, etc.
F12
PLG
KLKB1
SERPINF2
Fibrinolytic system
KNG
Bradykinin
BDKR
Inflammation, prostaglandin biosynthesis, nitric oxide biosynthesis, etc.
Complement cascade
HF1
Alternative pathway
Zymosan, inulin, etc.
BF
C3
DF
C3b/Bb
IF
CRRY
DAF
MCP
CD59
C6
C7
C8
C9
Lectin pathway
MBL
MASP1/2
C4b/2a
C3 Convertase
C3b
C5
C5b
Membrane attack complex
Cell lysis
Classical pathway
C2
C4
C4a
C3a
C5a
Antibody/ antigen complex
C1Q
C1R
C1S
C4BP
C3b/4b, C3b, C3d
SERPING1
Mediators
C3AR1
C5R1
CR2
CR1
Muscle contraction, chemotaxis, phagocyte recruitment, inflammation, etc.
B cell receptor signaling pathway
NS 1
NS 2A
NS 2B
NS 3
NS 5
E
M
04610 4/3/09
(c) Kanehisa Laboratories

q

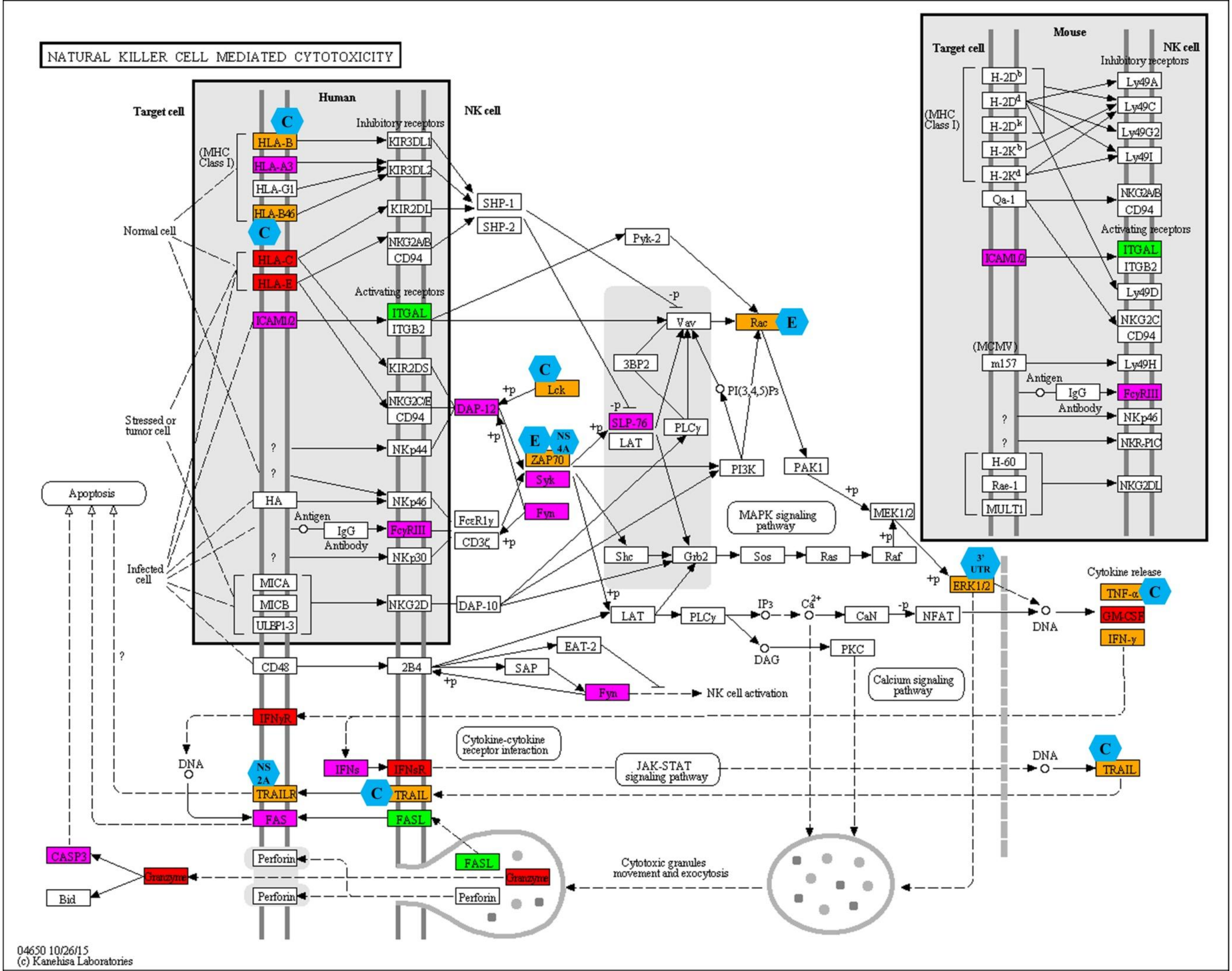

NATURAL KILLER CELL MEDIATED CYTOTOXICITY
Human
Target cell
NK cell
Inhibitory receptors
(MHC Class I)
HLA-B
HLA-A3
HLA-G1
HLA-B46
HLA-C
HLA-E
KIR3DL1
KIR3DL2
KIR2DL
NKG2A/B
CD94
SHP-1
SHP-2
Normal cell
Activating receptors
ICAM1/2
ITGAL
ITGB2
KIR2DS
NKG2C/E
CD94
DAP-12
Lck
+p
Stressed or tumor cell
NKp44
?
ZAP70
Syk
Fyn
E
NS 4A
C
HA
NKp46
FcεR1γ
Antigen
IgG
Antibody
FcγRIII
CD3ζ
NKp30
Infected cell
MICA
MICB
ULBP1-3
NKG2D
DAP-10
CD48
2B4
EAT-2
SAP
Fyn
NK cell activation
Pyk-2
Vav
-p
Rac
3BP2
SLP-76
LAT
PLCγ
PI(3,4,5)P3
PI3K
PAK1
MEK1/2
MAPK signaling pathway
Shc
Grb2
Sos
Ras
Raf
ERK1/2
3' UTR
LAT
PLCγ
IP3
Ca2+
CaN
NFAT
DAG
PKC
DNA
Cytokine release
TNF-α
GM-CSF
IFN-γ
Calcium signaling pathway
Apoptosis
IFNγR
DNA
IFNs
IFNsR
Cytokine-cytokine receptor interaction
JAK-STAT signaling pathway
DNA
TRAIL
NS 2A
TRAILR
TRAIL
FAS
FASL
FASL
CASP3
Granzyme
Bid
Perforin
Perforin
Granzyme
Perforin
Cytotoxic granules movement and exocytosis
Mouse
H-2D^b
H-2D^d
H-2D^k
H-2K^b
H-2K^d
Qa-1
Ly49A
Ly49C
Ly49G2
Ly49I
NKG2A/B
CD94
ICAM1/2
ITGAL
ITGB2
Ly49D
NKG2C
CD94
(MCMV)
m157
Ly49H
Antigen
IgG
Antibody
FcγRIII
NKp46
NKR-P1C
H-60
Rae-1
MULT1
NKG2DL
04650 10/26/15
(c) Kanehisa Laboratories

r

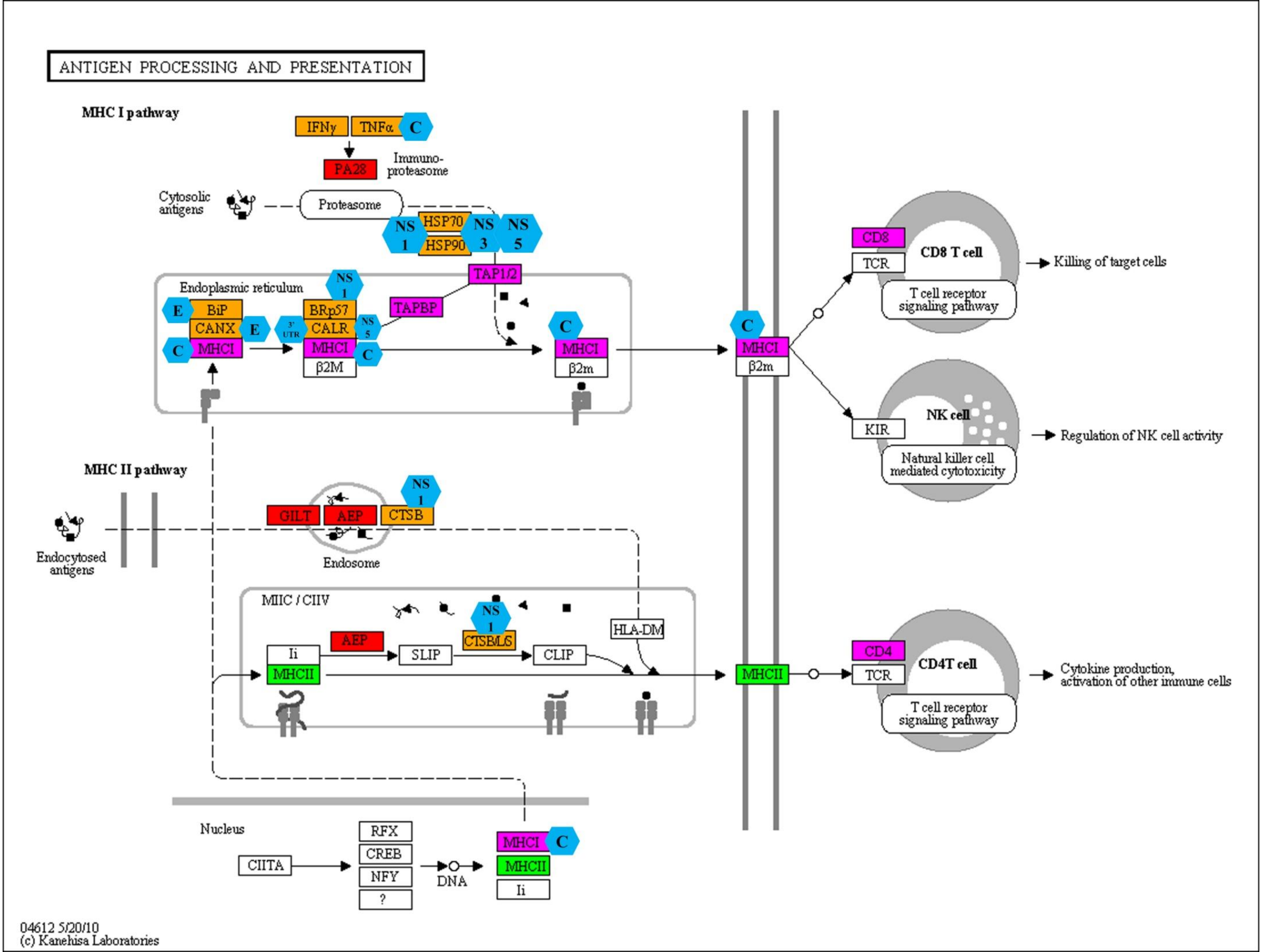
ANTIGEN PROCESSING AND PRESENTATION
MHC I pathway
IFNγ
TNFα
C
PA28
Immuno-
proteasome
Cytosolic
antigens
Proteasome
HSP70
HSP90
NS 1
NS 3
NS 5
TAP1/2
Endoplasmic reticulum
NS 1
E
BiP
CANX
E
C
MHCI
BRp57
CALR
3' UTR
NS 5
MHCI
C
β2M
TAPBP
C
MHCI
β2m
C
MHCI
β2m
CD8
TCR
CD8 T cell
T cell receptor
signaling pathway
Killing of target cells
NK cell
KIR
Natural killer cell
mediated cytotoxicity
Regulation of NK cell activity
MHC II pathway
GILT
AEP
CTSB
NS 1
Endocytosed
antigens
Endosome
MIIC / CIIV
NS 1
HLA-DM
Ii
MHCII
AEP
SLIP
CTSB/L/S
CLIP
MHCII
CD4
TCR
CD4T cell
T cell receptor
signaling pathway
Cytokine production,
activation of other immune cells
Nucleus
RFX
CREB
NFY
?
CIITA
DNA
MHCI
C
MHCII
Ii
04612 5/20/10
(c) Kanehisa Laboratories

S

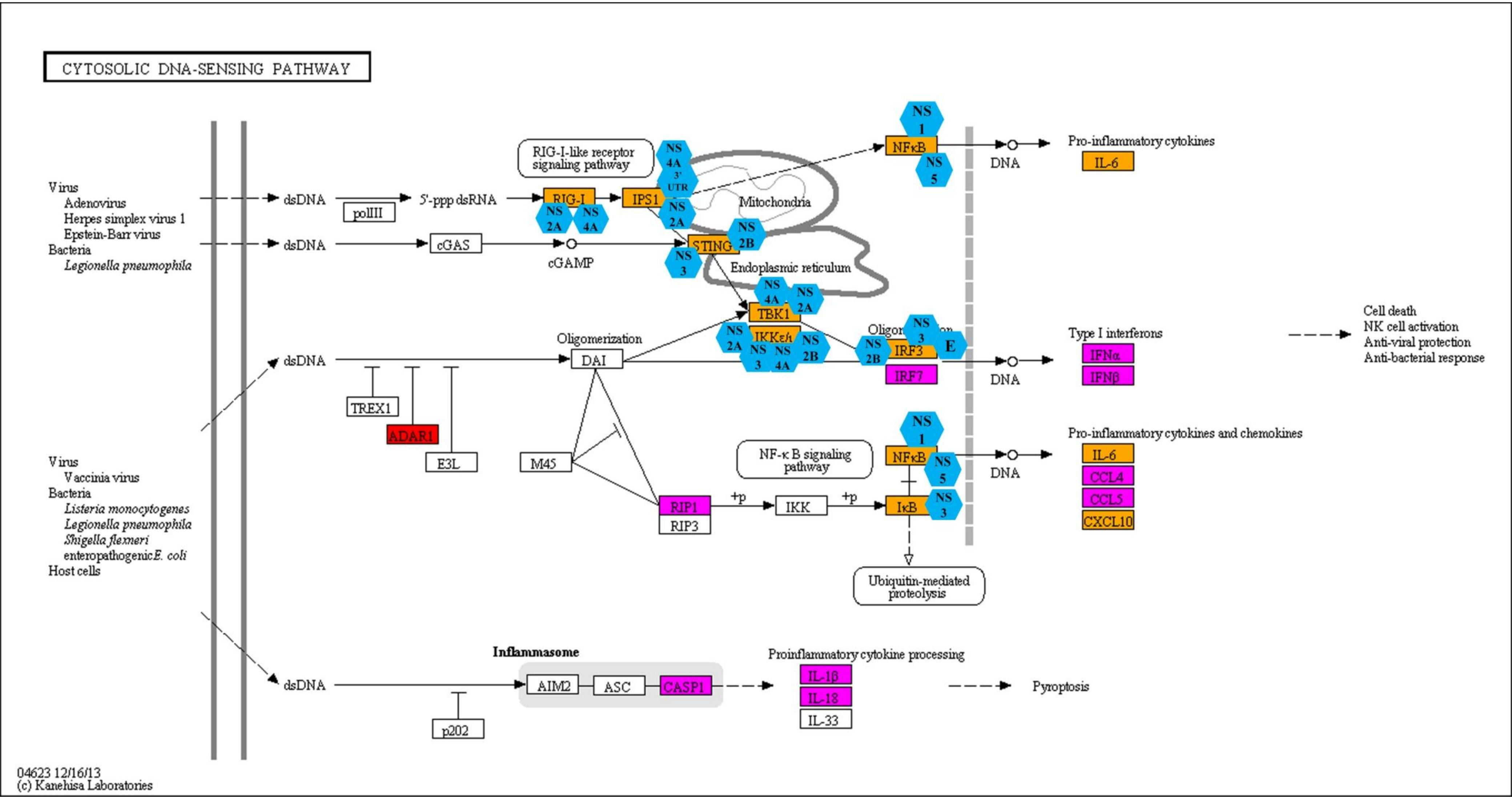
CYTOSOLIC DNA-SENSING PATHWAY
Virus
Adenovirus
Herpes simplex virus 1
Epstein-Barr virus
Bacteria
Legionella pneumophila
dsDNA
polIII
5'-ppp dsRNA
RIG-I-like receptor signaling pathway
RIG-I
IPS1
Mitochondria
cGAS
cGAMP
STING
Endoplasmic reticulum
TBK1
IKKε/i
Oligomerization
DAI
IRF3
IRF7
NFκB
DNA
Pro-inflammatory cytokines
IL-6
Type I interferons
IFNα
IFNβ
Cell death
NK cell activation
Anti-viral protection
Anti-bacterial response
TREX1
ADAR1
E3L
M45
Virus
Vaccinia virus
Bacteria
Listeria monocytogenes
Legionella pneumophila
Shigella flexneri
enteropathogenic E. coli
Host cells
NF-κB signaling pathway
RIP1
RIP3
+p
IKK
IκB
Ubiquitin-mediated proteolysis
Pro-inflammatory cytokines and chemokines
CCL4
CCL5
CXCL10
Inflammasome
AIM2
ASC
CASP1
p202
Proinflammatory cytokine processing
IL-1β
IL-18
IL-33
Pyroptosis
NS 1
NS 5
NS 4A
3' UTR
NS 2A
NS 2B
NS 3
E
04623 12/16/13
(c) Kanehisa Laboratories

t

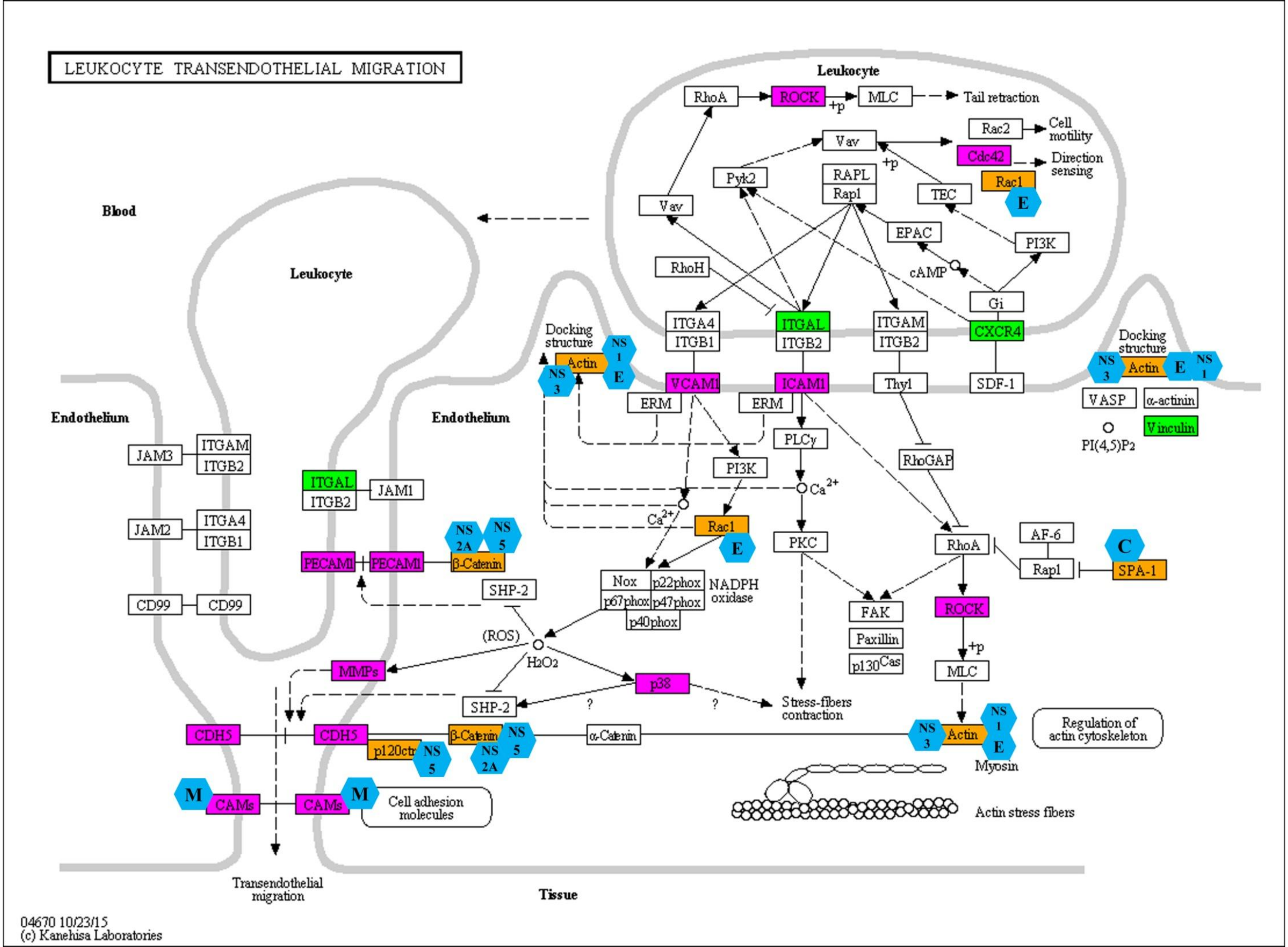

LEUKOCYTE TRANSENDOTHELIAL MIGRATION
Leukocyte
RhoA
ROCK
+p
MLC
Tail retraction
Rac2
Cell motility
Vav
Cdc42
Direction sensing
+p
Rac1
E
Pyk2
RAPL
Rap1
TEC
Vav
EPAC
PI3K
Blood
RhoH
cAMP
Gi
Leukocyte
ITGA4
ITGB1
ITGAL
ITGB2
ITGAM
ITGB2
CXCR4
Docking structure
Actin
NS 1
E
NS 3
VCAM1
ICAM1
Thy1
SDF-1
Docking structure
NS 3
Actin
E
NS 1
ERM
ERM
VASP
α-actinin
Vinculin
PI(4,5)P2
Endothelium
Endothelium
JAM3
ITGAM
ITGB2
PLCγ
ITGAL
ITGB2
JAM1
PI3K
RhoGAP
Ca2+
JAM2
ITGA4
ITGB1
Ca2+
Rac1
E
NS 2A
NS 5
PECAM1
PECAM1
β-Catenin
PKC
RhoA
AF-6
C
Rap1
SPA-1
SHP-2
Nox
p22phox
p67phox
p47phox
p40phox
NADPH oxidase
FAK
Paxillin
p130Cas
ROCK
CD99
CD99
(ROS)
H2O2
+p
MLC
MMPs
p38
SHP-2
?
?
Stress-fibers contraction
CDH5
CDH5
β-Catenin
NS 5
α-Catenin
NS 3
Actin
NS 1
E
Regulation of actin cytoskeleton
p120ctn
NS 5
NS 2A
NS 5
Myosin
M
CAMs
CAMs
M
Cell adhesion molecules
Actin stress fibers
Transendothelial migration
Tissue
04670 10/23/15
(c) Kanehisa Laboratories
