# Supplementary material for: DenHunt - A Comprehensive Database of the Intricate Network of Dengue-Human Interactions
Source: PLoS Negl Trop Dis. 2016 Sep 12;10(9):e0004965. doi: 10.1371/journal.pntd.0004965 (PMC5019383; doi:10.1371/journal.pntd.0004965)
Supplement: S1 Checklist — (DOC) [file pntd.0004965.s013.doc]

| **Section/topic** | **#** | **Checklist item** | **Reported on page # of the final revised paper.** |
| --- | --- | --- | --- |
| **TITLE** | | |  |
| Title | 1 | DenHunt - A Comprehensive Database of the Intricate Network of Dengue - Human Interactions. This is a meta-analysis leading to construction of a database called DenHunt | Pg.No. 1,  Line No. 1-2 |
| **ABSTRACT** | | |  |
| Structured summary | 2 | DENV is an emerging vector-borne human pathogen that affects millions of individuals each year by causing severe and potentially fatal syndromes (Dengue Hemorrhagic fever (DHF) and Dengue shock syndrome (DSS)). Despite intense research efforts, no approved vaccine or antiviral therapy is yet available. Significant efforts have been directed towards understanding the pathogenesis of dengue infection and many interactions between DENV and human proteins that have been reported in literature. In this study, we mined publicly available dengue–human interactions that have been reported in the literature into a database called DenHunt. We retrieved 682 direct interactions of human proteins with dengue viral components, 382 indirect interactions and 4120 differentially expressed human genes in dengue infected cell lines and patients. Data in DenHunt was used to draw network maps of human-dengue relationships and observed that the virus targets multiple host functional complexes of important cellular processes such as metabolism, immune system and signaling pathways suggesting a potential role of these interactions in viral pathogenesis. Around 7 percent of the dengue interacting human proteins were also involved in other infectious and non-infectious diseases. Tthe understanding that comes from such analyses could be used to design better strategies to counteract the diseases caused by dengue virus. The whole dataset has been catalogued in a searchable database, called DenHunt (http://proline.biochem.iisc.ernet.in/DenHunt/). | Pg. No. 1-2, Line No. 18-33 |
| **INTRODUCTION** | | |  |
| Rationale | 3 | Dengue, an emerging infectious disease, is presently the most common arboviral disease globally. Approximately 2.5 billion people live in dengue infested regions worldwide and 390 million dengue infections are reported per year. Dengue infection leads to complications ranging from mild dengue fever to more severe dengue hemorrhagic fever (DHF) and dengue shock syndrome (DSS). It is not fully understood why most patients clear dengue infections quickly without any complications, whereas others develop a potentially fatal vascular leakage syndrome or severe hemorrhages. Despite intense research efforts, no approved vaccine or antiviral therapy is yet available.  Regardless of the difficulties in in vivo dengue viral research, significant efforts have been directed towards understanding the pathogenesis of dengue infection using in vitro platforms, such as cell lines as well as clinical samples such as patient’s blood, peripheral blood mononuclear cells (PBMCs) and serum. This valuable information remains disseminated along with other published literature in databases essentially in PubMed, making it difficult and time-consuming for dengue viral researchers to access and utilize the information for detailed computational analysis. Hence, there exists an immediate need for generating a database that provides readily usable simplified data pertaining to dengue-human interactions by collating all the existing information in literature. Many databases have been generated for different pathogens that provide curated interactions between pathogen – host components. However, dengue-human interactions are poorly represented in these databases with interactions extracted only from a 26 publications. | Pg. No. 3-4, Line No. 48-85 |
| **Section/topic** | **#** | **Checklist item** | **Reported on page #** |
| Objectives | 4 | The objective of this study is to describe the development of the database, DenHunt, summarize its contents, demonstrate the complexity of the dengue-human protein interaction network and compare it with networks of proteins interacting with other pathogens or involved in other diseases. We show that the information from such databases can help in creating network maps of how the virus disrupts cellular signaling and immune system pathways. We also list known FDA approved drugs against the dengue virus interacting human proteins that are being used to treat various other diseases. | Pg. No. 4-5, Line No. 86-107 |
| **METHODS** | | |  |
| Protocol and registration | 5 | We have not used any particular review protocol but we have used two types of keyword search to fetch papers related to dengue human interactions from peer reviewed publications indexed in PubMed. The first type of query was using keywords in the S1 Table the second was using dengue protein names as queries. | Pg. No. 5-7, Line No. 109-144. |
| Eligibility criteria | 6 | Abstracts of the papers retrieved through keyword search were examined manually. The eligibility criteria for selecting a publication are if it describes any human protein to be important for dengue viral infection. | Pg. No. 5, Line No. 113-117 |
| Information sources | 7 | Our information resource is PubMed database. We have performed keyword search for papers published on dengue till 31-Oct-2015. | Pg. No. 8-9, Line No. 189-190 |
| Search | 8 | Details of the keyword search is given the methods section of the paper and below:   1. For the queries using the keywords in S1 Table, the search was carried out using the syntax “(Dengue[Title/Abstract]) AND keyword”. These keywords are usually used to describe protein-protein interactions and majority of them were retrieved from the publication that describes construction of the HIV-1 human protein interaction database “Ptak RG, Fu W, Sanders-Beer BE, Dickerson JE, Pinney JW, Robertson DL, et al. Cataloguing the HIV type 1 human protein interaction network. AIDS Res Hum Retroviruses. 2008;24(12):1497-502. doi: 10.1089/aid.2008.0113. PubMed PMID: 19025396”. Search was carried out using all forms of the keywords: for eg. Phosphorylated, phosphorylating and phosphorylation. 2. We also performed searches using all the dengue proteins as queries: For. Eg. “Dengue NS1”. | The workflow chart is given in the Checklist S2: PRISMA Flowchart and the methods section of the final paper: Pg. No. 5-7, Line No. 109-144. |
| Study selection | 9 | All abstracts were selected if they describe a human protein to be important for dengue infection. Then, full length papers are read to classify the publications into those that describe direct dengue-human protein interactions, indirect or functional analysis and differentially expressed genes in dengue infection. If any predicted interactions have been reported, we selected only those that have been shown to be associated with dengue infection in previous publications. | Pg. No. 9, Line No. 192-197 |
| **Section/topic** | **#** | **Checklist item** | **Reported on page #** |
| Data collection process | 10 | The full length paper of the selected retrieved publications was examined to extract information regarding dengue – human interactions.  **Gene Symbol and Entrez gene id:** Most papers describe proteins as their gene synonym. We have extracted the official gene symbol and entrez gene id was retrieved from bioDBnet (http://biodbnet.abcc.ncifcrf.gov/), GeneCards (http://www.genecards.org/) and Synergizer (http://llama.mshri.on.ca/synergizer/translate/).  **System used:** The methods and results section was examined to extract information of which experiment was used to detect the interaction.  **Serotype or Patient type:** The methods section was examined to extract information of which experiment was used to detect the interaction.  **Comparison:** The methods and results section was examined to extract this information..  **Variation:** The results section was examined to extract this information. | Pg. No. 6, Line No. 124-144 |
| Data items | 11 | The different data items extracted were: Dengue viral component, Human Gene symbol, Human gene Entrez ID, Pubmed ID, Patient type or Serotype, System, Comparison and Variation. | Pg. No. 6, Line No. 124-144 |
| Risk of bias in individual studies | 12 | Certain experimental techniques such as microarray and proteomic analysis have a high false discovery rate. So to eliminate this bias in all downstream pathway analysis, we selected differentially expressed genes that were consistently up or down regulated in dengue infection in 4 publications. | Pg. No. 7, Line No. 156-159 |
| Summary measures | 13 | - | - |
| Synthesis of results | 14 | **Data curation and generation:** We obtained the our consolidated data in the following manner; (1) keyword search, (2) manual curation to select publications that describe human protein that are important in dengue infection, (3) classification of data into the three classes, and (4) extraction of the different data items from the publications.  **KEGG normal pathway and disease pathway analysis:** In order to find enriched KEGG normal and disease pathways we used a tool (WebGestalt) that extracts KEGG pathways that are statistical significant using hypergeometric test and the P-value was adjusted by the Benjamini & Hochberg (BH) method. Only pathways that have a adjusted p-value less than 0.01 are selected.  **Dengue interactome as a source of drug repurposing:** We extracted a subset of dengue interacting human proteins that have been shown to be important for dengue viral replication (we call them dengue virus host dependency factor, DVHF) as inhibiting these protein by various experimental methods led to an reduction in dengue viral replication. We then extracted FDA drugs that showed pharmacological action against these proteins and being used to treat other diseases. | Pg. No. 5-8, Line No. 108-185 |

| **Section/topic** | **#** | **Checklist item** | **Reported on page #** |
| --- | --- | --- | --- |
| Risk of bias across studies | 15 | Since the data extracted was from peer reviewed publications, all the selected data has been included in the database. | Pg. No. 6, Line No. 118-120 |
| Additional analyses | 16 | - | - |
| **RESULTS** | | |  |
| Study selection | 17 | Using the keyword search, we retrieved 6576 unique publications. After screening of all the retrieved papers abstracts, we selected 287 publications. All the information of the dengue – human interaction was extracted from the methods and results section of the publications. The data extracted from these publications was used to construct the database and for all our downstream analysis. | Pg. No. 9, Line No. 198-200 |
| Study characteristics | 18 | For each interaction, we extracted the following data items:  **Dengue viral component:** The dengue viral component involved in the interaction. This field is available only for the direct interactions.  **Human Gene symbol:** The official gene symbol of the human protein interactor.  **Human gene Entrez ID:** The gene identification numbers from Entrez Gene, NCBI's database for gene-specific information.  **Pubmed ID:** PMIDs of articles describing the interaction.  **Patient type or Serotype:** The serotype of the DENV strain or strains (DENV1, 2, 3, 4) used in the study. If clinical samples from patients such as serum, whole blood or peripheral blood mononuclear cells (PBMCs) are used, then type of patient (DF, DHF and DSS) used in the study is given.  **System:** This field gives information of the cell line used in the study if it is an in vitro study. If the study uses clinical samples, then the type of sample used such as serum, whole blood or peripheral blood mononuclear cells (PBMCs) is given.  **Comparison:** This field is available only for differentially expressed genes where the expression of genes in infected samples is compared with controls. eg. Dengue infected 293T cells vs uninfected 293T cells)  **Variation:** This field is available only for differentially expressed interactions. It states whether the gene is up regulated or down regulated in dengue infected samples. | Pg. No. 6-7, Line No. 124-144 |
| Risk of bias within studies | 19 | - | - |
| Results of individual studies | 20 | - | - |
| **Section/topic** | **#** | **Checklist item** | **Reported on page #** |
| Synthesis of results | 21 | **Data curation:** There are around 14,559 publications describing dengue viral research in PubMed till 31 October 2015. Literature describing human proteins important for dengue infection was extracted from PubMed by queries using the keywords listed in S1 Table and dengue proteins.  The retrieved interactions were classified into three types: (i) Direct interactions, where the human proteins physically interact with the viral proteins or RNA, (ii) Indirect or functional interactions, where the human proteins affect viral replication but there exists no current evidence of them directly interacting with the viral components and (iii) Differentially expressed interactions, genes or proteins whose expression patterns are altered during dengue viral infection.  We identified 682 direct, 382 indirect and 4120 differentially expressed interactions from 103, 151 and 41 references in PubMed respectively. A database called DenHunt was constructed using this data. The data can be downloaded from S2 Dataset and download section of the database. | Pg. No. 8-12, Line No.187-263 |
|  |  | **Pathway analysis:** We carried out pathway enrichment analysis using the online tool WebGestalt and selected only pathways that had at least 3 proteins and an adjusted p-value ≤ 0.01. We have seen an enrichment of pathways of signalling, immune, metabolism, transport and catabolism in the dengue virus interacting human proteins. | Pg. No. 12 - 15 Line No. 264-338 |
|  |  | **Asociation of dengue – interactome with disease pathways:** We have also seen many disease pathways enriched in our gene list. 273 dengue interacting human proteins are associated with other infectious diseases and 168 of them are associated with more than one infectious disease. 249 dengue interacting human proteins are associated with non-infectious diseases and 135 of them are associated with more than one non-infectious disease. We also observed 140 dengue interacting human proteins associated with both infectious and non-infectious diseases. This shows that many human proteins of dengue-human interactome are also involved in the response to pathogenic infections and other complex non-infectious diseases. | Pg. No. 16-17, Line No. 339-382 |
|  |  | **Dengue-Human interactome, a potential resource for drug repurposing:** 263 dengue virus interacting proteins were considered to be potential DVHFs because they were described to be essential for viral replication in publications, as inhibiting these proteins led to a reduction in viral replication. 20 of the 263 DVHFs had known FDA approved drugs targeted against them and 9 of these DVHFs were associated with more than one infectious or non-infectious disease. Two of the proteins CCR5 and HMGCR are already established drug targets against another virus, HIV. Maraviroc and lovastatin, inhibitors of CCR5 and HMGCR respectively, have already been shown to inhibit dengue viral replication in in vitro studies.These drugs could be tested for their anti-dengue viral effect and it is possible that some of these drugs, either singly or in synergistic combinations may prove to be effective antiviral agents. | Pg. No. 17-20, Line No. 383-431 |
| Risk of bias across studies | 22 | - | - |
| Additional analysis | 23 | - | - |
| **Section/topic** | **#** | **Checklist item** | **Reported on page #** |
| **DISCUSSION** | | |  |
| Summary of evidence | 24 | DenHunt was developed as a user friendly public repository to capture and organize manually curated information from the available scientific literature on the interactions between dengue virus and host proteins. DenHunt could be used to make detailed maps tracking cellular interactions that drive dengue viral replication, and provides a discovery space to the research community for researching and better understanding the dengue viral pathogenesis. Our pathway analysis section shows how dengue virus targets multiple components of the same pathways to mediate effects such as apoptosis or inhibition of IFNα/β production. Although the approach adopted here is purely qualitative, we have amply demonstrated how an integrated repository such as DenHunt could be used to harness already existing data to elucidate dengue viral pathogenesis mechanisms. The key to gain new understanding from DenHunt in viral pathogenesis would lie in its integration with other sources of multidimensional data such as time-course dengue infected gene expression data. | Pg. No. 20-21 Line No. 432-469 |
| Limitations | 25 | - | - |
| Conclusions | 26 | We have developed a consolidated dengue virus-human interactome database called DenHunt, which contains a compilation of a curated set of experimentally verified dengue-human interactions. Detailed characterization of the relationships between these interactions that include multidimensional data given in the database such as direct physical interactions, indirect interactions, gene expression patterns, gene silencing studies, virus serotype, cell type, disease stage, etc., will lead to improved understanding of the conflict between dengue and its human host. | Pg. No. 21-22 Line No. 470-479 |
| **FUNDING** | | |  |
| Funding | 27 | - | - |

*From:*  Moher D, Liberati A, Tetzlaff J, Altman DG, The PRISMA Group (2009). Preferred Reporting Items for Systematic Reviews and Meta-Analyses: The PRISMA Statement. PLoS Med 6(7): e1000097. doi:10.1371/journal.pmed1000097

For more information, visit: **www.prisma-statement.org**.

Page 2 of 2
