# Supplementary material for: DenHunt - A Comprehensive Database of the Intricate Network of Dengue-Human Interactions
Source: PLoS Negl Trop Dis. 2016 Sep 12;10(9):e0004965. doi: 10.1371/journal.pntd.0004965 (PMC5019383; doi:10.1371/journal.pntd.0004965)
Supplement: S1 Flowchart — (DOC) [file pntd.0004965.s014.doc]

**Flow Diagram of the workflow**

**Screening**

**Included**

**Eligibility**

**Identification**

Records identified through Pubmed database searching
(n = 14,703)

Additional records identified through other sources
(n = 0)

Records after duplicates removed
(n = 6576)

Abstracts of records screened
(n = 6576)

Records excluded with reasons
(n = 6289)

Full-text articles assessed for eligibility
(n = 287)

Full-text articles excluded, with reasons
(n = 0 )

Studies included in quantitative synthesis (meta-analysis and database)
(n = 287)
